# Digital infrastructure and proxies of ambulatory care access in Russia, 2018–2024: a regional panel study with a national telemedicine signal analysis

**DOI:** 10.3389/fdgth.2026.1856577

**Published:** 2026-06-23

**Authors:** Artem Bobkov, Tatiana Bobkova, Liuying Wang, Nikita Sokolov, Jinpeng Xu, Fangmin Deng, Valery Royuk, Zheng Kang

**Affiliations:** 1 School of Health Management, Harbin Medical University, Harbin, China; 2 Research and Educational Resource Center for Immunophenotyping, Digital Spatial Profiling and Ultrastructural Analysis Innovative Technologies, RUDN University, Moscow, Russia; 3 Institute of Leadership and Health Management, Federal State Autonomous Educational Institution of Higher Education I.M. Sechenov First Moscow State Medical University of the Ministry of Healthcare of the Russian Federation (Sechenovskiy University), Moscow, Russia

**Keywords:** ambulatory care, digital health, digital infrastructure, healthcare access, measurement sensitivity, regional panel study, Russian Federation, telemedicine

## Abstract

**Background:**

In the present study, telemedicine volume is considered in a bounded measurement sense: as an administratively registered count of remote care activity whose relationship to access expansion requires separate assessment. Its interpretation remains uncertain in a territorially uneven environment, where territorial digital readiness and the resource configuration of the ambulatory sector shape the infrastructural-resource context, while the reporting regime affects the composition of the registered flow. The study assessed regional proxies of ambulatory contact and structural capacity; the national Russian Federation–year series was examined separately as a measurement signal sensitive to the composition of the reported flow and its temporal stability.

**Methods:**

An observational regional panel study used official data for 85 Russian territories in 2018–2024, comprising 595 region–year observations. Sources were Rosstat/EMISS and Form No. 30. Outcomes were physician visits per 1,000 population, treated as the realized contact intensity (RCI) proxy, and ambulatory care organization (ACO) capacity per 10,000 population, treated as the structural capacity of ambulatory care (SCA) proxy; both were aggregated regional proxies, without extrapolation to individually realized access. The digital environment was measured using the Digital Infrastructure Index (DII). Analyses included descriptive diagnostics, correlations, two-way fixed effects (TWFE) models, and first-differences (FD) models; the national series was analyzed separately for measurement sensitivity.

**Results:**

Digital-environment associations with ambulatory indicators were expressed mainly interregionally. The DII–physician visit association changed from +0.258 in the pooled specification to −0.031 after two-way centering; the association with ACO capacity shifted from +0.087 to −0.003. The national telemedicine series increased from 0.39–0.68 million consultations in 2018–2019 to 7.00 million in 2020 and 15.94 million in 2024. The patient–physician share reached 0.919 in 2020 and declined to 0.496 in 2024.

**Conclusions:**

In the Russian regional context, growth in telemedicine volume is insufficient to interpret telemedicine as an autonomous mechanism for equalizing access. It should be read as a bounded monitoring signal: territorial digital context and ambulatory-sector resource configuration define interpretation, while contact modality and the comparability of reporting definitions impose further inferential limits. Digital health monitoring should link telemedicine counters to patient-pathway segments, distinguishing remote-channel scaling from factual expansion of realized access.

## Graphical Abstract

**Figure:**
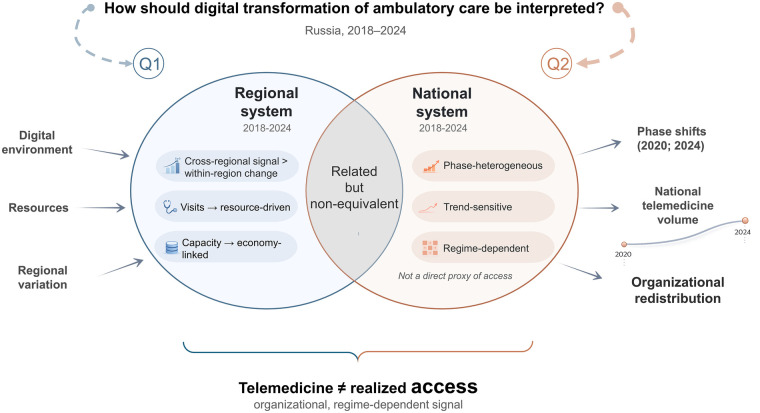

## 1 Introduction

In the present study, telemedicine volume (TM) is used in a bounded measurement sense as an administratively registered count of remote activity, generated within reporting and insurance procedures and information systems in which the fact of contact may be captured through interaction modality and service code; the inclusion of a record in a particular registered flow defines its position within the administrative contour (1, 2). This administrative visibility characterises service activity and the workload imposed on the reporting contour, while leaving open the question of whether the recorded contact became part of a patient pathway that was factually traversed. Therefore, the interpretation of telemedicine volume requires a separate alignment of the registered contact with access and the quality of remote service delivery; patient experience and the effectiveness of the digital scenario constitute an additional level at which this signal must be assessed (3, 4). Remote formats can support continuity of care during periods of systemic shock; however, high telemedicine intensity in large insurance-based datasets is associated with changes in the overall contact burden, including the dynamics of ambulatory care-sensitive hospitalisations and expenditures, which renders its system-level meaning analytically ambiguous (5, 6). Additional uncertainty is introduced by the modal heterogeneity of the telemedicine flow: post-pandemic evidence demonstrates a pronounced divergence between video and audio formats, while expanded availability of the remote channel in primary care may be accompanied by a redistribution of contacts without a marked increase in their aggregate volume (7, 8). Within this framework, telemedicine volume is meaningful as a signal of changes in entry into the healthcare system; any conclusion about the expansion of realised access requires verification of how the registered contact corresponds to the patient's movement from emergent need to care actually received.

Methodological caution in interpreting TM volume is further reinforced by the fact that the remote channel develops within an already stratified digital environment, where the attainability of remote contact depends on the territorial profile and the patient's social resources (9). This dependence is expressed in the persistent association of telemedicine use with rural territorial profiles and broadband (BB) infrastructure: large empirical datasets show that the remote channel is used less evenly where spatial remoteness coincides with limited digital connectivity (10, 11). Territorial deprivation further complicates the interpretation of digital entry, since the probability of seeking remote care and the choice of modality are unevenly distributed across patient groups; telephone or audio-only formats are more frequently associated with age and language- or technology-related constraints (12–14). Evidence on the sensitivity of telepsychiatry implementation to patients' place of residence (15–17) supports a broader inference: the growth of remote contacts must be read through the unequal traversability of the digital channel across territories and patient groups, and its distributional meaning therefore requires a more cautious interpretation than a linear reading of access equalisation.

Even where territorial digital infrastructure is well developed, the remote care-delivery channel requires an intermediate implementation layer through which technical feasibility is converted into a stable format of care receipt. Empirical evidence from territories beyond large metropolitan agglomerations shows that the association between telemedicine and quality indicators depends on the local organisational configuration (18). This logic is further specified in the literature on telemedicine implementation: sustained use of the remote format emerges where readiness and needs assessment is translated into managed change planning under organisational leadership, while subsequent monitoring keeps the intervention embedded in the workflow (19–21). On the patient side, the traversability of the digital scenario is determined by the extent to which the service remains usable and trustworthy, and by whether the implementation of digital tools accounts for the risk of reproducing inequality (22, 23). The Digital Infrastructure Index (DII) should therefore be treated as a characteristic of the regional digital context, analytically separated from organisational readiness, patient acceptance of telemedicine, and completed remote use of care. Its association with observed indicators of ambulatory contact requires interpretation through the organisational and user-side traversability of the remote care-delivery channel.

Within the Russian context, this problem acquires independent analytical significance because telemedicine develops within a state-centred digital architecture in which remote services are embedded in centralised information exchange and regional medical information systems (24, 25). The institutional trajectory of this architecture can be traced back to the establishment of the Unified State Information System in Healthcare (EGISZ) in 2011; since 2019, the development of an EGISZ-based unified digital healthcare contour has been incorporated into a federal project, within which electronic medical documentation and digital services became part of managed interorganisational interaction (26). The legal codification of telemedicine has evolved from a period of incomplete federal regulation in 1998–2017 to a basic procedure governing the use of telemedicine technologies and, after 2020, was supplemented by experimental legal regimes; the dependence of remote treatment adjustment on a prior in-person encounter preserves a restricted logic of digital entry into the healthcare system (24). TM growth within this configuration requires a regime-sensitive reading: in 2021, the number of consultations increased by 23% compared with 2020, the share of consultations financed through compulsory health insurance rose from 6.95% to 11.72%, the number of patients under remote monitoring increased by 44%, and the 2022 tariff agreements recorded an expansion in the number of telemedicine service types in seven regions, alongside a reduction in ten regions (27). Digitalisation of healthcare in Russia remains heterogeneous at the level of practical implementation: the development of medical information systems depends on the legal regime and the digital readiness of healthcare workers, while on the patient side, trust in the remote format constitutes an additional condition for the traversability of the digital scenario (28). International comparison supports this reading: in the United States, the share of telemedicine among physician visits shifted from less than 0.05% in 2019 to 25% in April 2020% and 4% in March 2023, demonstrating the sensitivity of telemedicine series to payment rules and the territorial profile of the system (29). Under this organisation of measurement, the Russian national telemedicine series should be read as a service-administrative signal in which contact volume is linked to the legal regime and the digital contour. The regional tariff configuration further limits its direct use as an indicator of access equalisation; the relationship between registered volume and care actually realised therefore requires separate assessment (30).

This logic defines the research gap addressed in the present study: the empirical interpretability of telemedicine counts formed within official reporting remains uncertain in relation to access to ambulatory care when individually realised access and the aggregated registration of medical activity belong to different levels of observation. The methodological basis for this formulation is that unmet need and actual healthcare utilisation describe distinct measurement planes of access, requiring joint interpretation while preserving the analytical distinction between them (31). Within the telemedicine contour, additional uncertainty arises at the level of remote-contact registration: visit modality may depend on the structure of electronic scheduling and billing capture, while the comparability of administrative data is determined by coding rules and the timeliness of data updating (32, 33). Within this framework, the present study assesses the association of the regional digital environment and healthcare resource endowment with physician visits per 1,000 population as the RCI proxy; ACO capacity per 10,000 population serves as the second regional outcome and represents the SCA proxy. The national telemedicine series is examined separately as a measurement signal whose interpretability depends on the stability of the modality structure and on the reporting regime within which the composition of the registered flow may change over time.

## 2 Materials and methods

### 2.1 Study design and analytical framework

The study was conducted as an observational analysis of the region–year panel, using annual data for the constituent entities of the Russian Federation over 2018–2024, and was guided by a preregistered study protocol deposited in the Open Science Framework (OSF) (34).

The design was structured through an explicit separation of analytical levels: the regional digital context was examined in relation to the ambulatory care contour, while the national telemedicine series retained an independent measurement role. The region–year panel was used to assess how the regional digital context and healthcare resource endowment were associated with aggregated proxies of ambulatory contact and ambulatory-sector structural capacity.

The unit of observation was the region–year observation, which enabled within-territory change over time to be examined while accounting for the calendar structure of the data and stable interregional differences. The RF–year telemedicine series was constructed separately from official reporting on the use of telemedicine technologies; because it had no regional variation, it was not included in the regional models and was treated as a macro-level signal through which the directionality of the telemedicine series, its temporal sensitivity, and its measurement stability were assessed under changes in the composition of the registered flow and the reporting regime. Individually realised access was treated as an adjacent level of interpretation, while episode completion and unmet need marked additional boundaries for interpreting the findings. The empirical contour of the article was therefore confined to aggregated regional proxies and the national measurement signal.

### 2.2 Data sources and panel construction

The analysis was based on secondary processing of official statistical data from the Russian Federation, including annual Rosstat/EMISS series and Form No. 30 reporting, Section 7004. After standardising the original data exports, harmonising territorial and temporal identifiers, and verifying the level of aggregation, a region–year panel covering 85 territories was assembled, comprising 595 region–year observations. A unified territorial grid of 89 constituent entities of the Russian Federation served as the reference framework. Four territories—the Donetsk People's Republic, the Luhansk People's Republic, Kherson Oblast, and Zaporizhzhia Oblast—were not included in the analytical dataset because comparable regional coverage was unavailable in the source data. Their exclusion was therefore required to preserve a consistent and comparable territorial basis for the region–year panel.

Only territorial units that were comparably represented across all data sources after harmonisation to the unified territorial grid were retained in the analytical dataset. Observations with a non-comparable level of aggregation, or without the possibility of unambiguous reduction to the regional level, were excluded. The RF–year series was constructed separately. A detailed inventory of the source tables is provided in Supplementary Data Sheet S1.

### 2.3 Outcomes, covariates, and variable operationalization

The primary regional outcome was the number of physician visits across all specialties per 1,000 population, used as the RCI proxy. Within the analytical framework of the study, this indicator captured aggregated ambulatory-sector contact activity at the regional level and was not extrapolated to individually realised access. Its interpretation was confined to the observed fact of physician contact in regional statistics, whereas episode completion and unmet need represented adjacent levels of access that were outside the direct measurement scope of this outcome. Telemedicine consultations were treated within a separate measurement contour: comparable indicators were available at the RF–year level and therefore did not constitute an independent regional component of the RCI proxy, being analysed instead as the national telemedicine signal.

The second regional outcome was ACO capacity per 10,000 population, operationalised as the SCA proxy. In this operationalisation, the ambulatory care contour was treated as the regional level of care organization most proximate to telemedicine consultations, because the remote format is functionally linked to physician contact and subsequent patient follow-up within the ambulatory logic of care. This indicator characterised the standardised organisational capacity of the ambulatory network within the regional care-delivery contour. The inpatient sector, including hospital bed capacity, belonged to a different level of resource structure; the spatial distribution of medical organizations and composite indexing were likewise outside the scope of this outcome.

Absolute indicators were converted into standardised rates according to the following formula:

$$x_{it}^{rate}=\frac{x_{it}}{Pop_{it}}\times s,$$

where $x_{it}$ denotes the absolute value of the indicator in region i in year t, $Pop_{it}$ denotes population size, and s is the scaling coefficient (1,000, 10,000, or 100,000).

Covariates included physician supply, hospital bed capacity, demographic rates, gross regional product (GRP) per capita, and indicators of the digital environment; logarithmic transformation was applied to positively skewed variables, including GRP per capita and the tariff indicator.

DII was calculated as the mean of available z-standardised components describing regional digital infrastructure and conditions of Internet access. The infrastructure block captured fixed broadband availability (fixed BB) and mobile broadband availability (mobile BB). The population block characterised household Internet access and Internet use by the population. The tariff component was represented by the cost of an Internet subscription and entered the index with a negative sign, so that higher DII values consistently corresponded to a more favourable digital environment. Within the analytical framework of the study, DII characterised territorial infrastructural readiness and the population-level digital context relevant to digitally mediated ambulatory access. Its interpretation was confined to the regional level: healthcare organizations' digital adoption capability and workforce engagement belonged to a different analytical layer, while patient trust in the remote format and actual use of telemedicine services defined the user-side and service-level contours through which the digital signal should be read.

For each component *k*, standardization was defined as:

$$z_{kit}=\frac{x_{kit}\;-\;\mu_{k}}{\sigma_{k}},$$

where $x_{kit}\;$ is the value of component k, and $\mu_{k}$ and $\sigma_{k}\;$ are the mean and standard deviation of the corresponding component in the analytical dataset. The index value was then calculated as:

$$DII_{it}=\frac{1}{K_{it}}\overset{K_{it}}{\underset{k=1}{\sum }}\;z_{kit},$$

where $K_{it}$ is the number of available components in a given observation; the index was calculated only when at least three components were available, in order to preserve minimum comparability across region–year observations while retaining sufficient analytical coverage.

TM indicators, available only at the RF–year level, included the total number of telemedicine consultations, the number of patient consultations, and their share in the overall telemedicine flow; all were normalised per 1,000 population of the Russian Federation. Full variable operationalization tables are presented in Supplementary Data Sheet S2.

### 2.4 Descriptive statistics, data quality assessment, and missing-data analysis

After the analytical datasets had been assembled, descriptive statistics, data quality assessment, and missing-data analysis were conducted before correlation diagnostics and regression modelling. The dataset passport, the expected size of the formally balanced region–year panel, completeness of coverage across regions and years, and the mean, standard deviation, median, interquartile range, and value range for all numerical variables were calculated. Missingness was assessed by variable and year, while extreme values were flagged within each year for verification against the source tables, without automatic exclusion. Only complete observations for the variables included in a given model were used in the estimations. This approach was chosen to preserve comparability across adjacent model specifications and to avoid introducing additional assumptions into the main analytical framework. The panel passport and the distribution of missingness are reported in Supplementary Data Sheet S3.

### 2.5 Correlation diagnostics and assessment of multicollinearity

Before estimating the panel models, correlation diagnostics were performed for selected pairs of digital indicators and outcomes. Pooled correlations were calculated to assess cross-regional co-variation, whereas correlations computed after two-way centering by region and year were used to evaluate within-region dynamics.

Partial correlations were estimated while controlling for regional economic level, physician supply, mid-level medical personnel supply, and the number of hospital organizations per 100,000 population; in the pooled specifications, year effects were additionally included. Multicollinearity was assessed using the variance inflation factor (VIF) and the condition number.

### 2.6 Statistical analysis: main panel models

The main analysis was conducted using a TWFE model:

$$Y_{it}=\beta^{⊤}X_{it}+\mu_{i}+\lambda_{t}+\varepsilon_{it},$$

where $Y_{it}$ denotes the outcome in region i in year t, $X_{it}$ is the vector of region-time predictors, and $\mu_{i}$ and $\lambda_{t}$ represent region and year fixed effects, respectively.

Models including resource predictors, DII, and individual digital components were considered; lagged models and FD models were used in the sensitivity analyses. The specification that included DII, resource indicators, and socioeconomic measures was treated as the main specification. Estimation was performed with robust standard errors clustered at the regional level. The full list of specifications is provided in Supplementary Data Sheet S4.

### 2.7 Secondary contextual analysis: the national telemedicine signal at the RF–year level

The RF–year series of telemedicine indicators was analysed as a separate measurement-sensitivity analysis block, positioned outside the regional models. Its function was to assess whether the national telemedicine signal preserved its directionality and temporal stability as a macro-level monitoring signal under changes in temporal specification. The assessment compared the baseline time-series description with trend-adjusted models; an additional sensitivity layer was introduced through first differencing and the exclusion of years marked by pronounced phase shifts. Within this framework, national TM volume was treated as a measurement series for evaluating the stability of the statistical signal under changes in the composition of the registered flow.

For each “telemedicine indicator × outcome” pair, ordinary least squares (OLS) models were estimated sequentially in levels:

$$M0:\;Y_{t}=\alpha +\beta TM_{t}+\varepsilon_{t},$$

in levels with a linear trend:

$$M1:\;Y_{t}=\alpha +\beta TM_{t}+\theta t+\varepsilon_{t},$$

and in first differences:

$$M3:\;\Delta Y_{t}=\alpha +\beta \Delta TM_{t}+\eta_{t},$$

where $Y_{t}$ denotes the outcome in year t, and $TM_{t}$ the telemedicine indicator.

Given T=7 and the presence of pronounced phase shifts in 2020 and 2024, the results were interpreted descriptively, with emphasis on sensitivity to trend adjustment, first differencing, exclusion of 2024, and sequential leave-one-year-out checks. The full model registry is provided in Supplementary Data Sheet S5.

### 2.8 Robustness and sensitivity analyses

Robustness checks were organised along three axes: variation in model form, variation in sample composition, and variation in the statistical inference scheme. The first axis comprised alternative one-way fixed-effects models, lagged specifications, and FD models estimated in the following form:

$$\Delta Y_{it}=\;\beta^{⊤}\Delta X_{it}+u_{it}$$

The second axis included exclusion of 2020, exclusion of 2020–2021, exclusion of the federal cities, and trimming of the distributional tails. The third axis encompassed sequential exclusion of individual regions and comparison of robust standard errors with alternative estimation schemes. A result was considered robust if it preserved its direction and a comparable order of magnitude across conceptually adjacent specifications. The full registry of robustness scenarios is presented in Supplementary Data Sheet S6.

### 2.9 Reproducibility, software, and data availability

Data preparation, construction of the region–year panel, and statistical modelling were carried out in a reproducible computational environment using Python 3.12.1, R 4.3.1, and Microsoft Excel (v. 2108, Build 14332.20721). The stages of standardisation, derivation of secondary indicators, dataset assembly, and model estimation were documented separately. The original data exports, standardised tables, intermediate analytical layers, and final results were preserved without manual alteration of values. A detailed inventory of software components, environment versions, and analytical parameters is provided in Supplementary Data Sheet S7.

## 3 Results

### 3.1 Data architecture, panel quality, and measurement constraints

**Figure 1:**
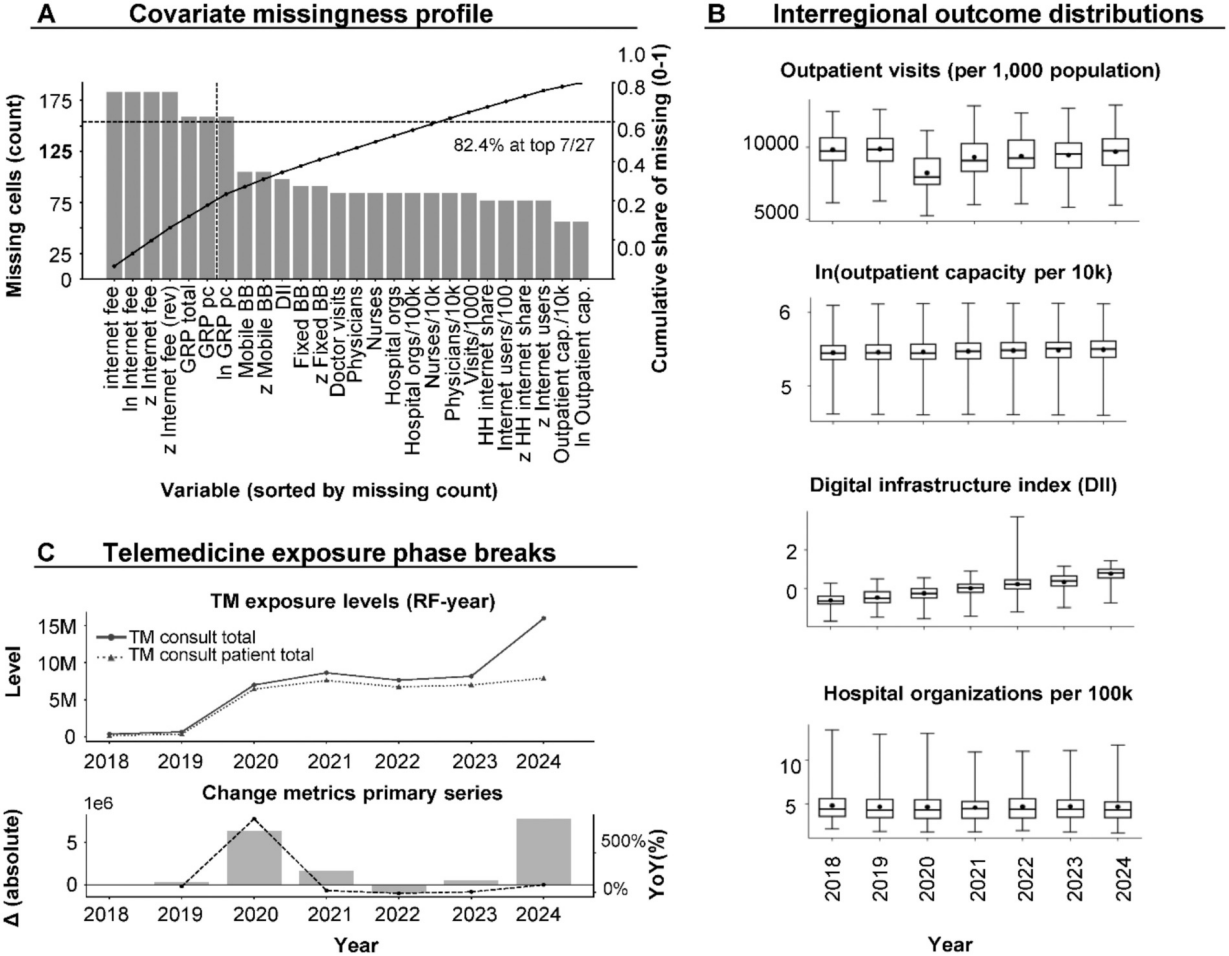
Data architecture and measurement constraints. **(A)** Missingness profile across covariates in the region–year panel; missing values are unevenly distributed and concentrated in a limited number of variables. **(B)** Annual cross-regional distributions of selected outcomes and resource indicators. Each subpanel uses its own *y*-axis corresponding to the indicator named in the heading; the scales are not intended for direct comparison across subpanels. **(C)** TM levels at the RF–year level and their annual changes [Δ and year-over-year (YoY) change].

Missingness was unevenly distributed and concentrated in a limited set of variables (Figure 1A). The largest losses were observed for Internet access tariff indicators, with 86 missing values out of 595 region–year observations (14.45%), and for GRP indicators, with 85 out of 595 missing values (14.29%); these were followed by mobile BB indicators, with 42 missing values out of 595 observations (7.06%). For most of the remaining indicators, incompleteness remained low: for ACO capacity, both in its original and logarithmic forms, missingness amounted to 7 out of 595 observations (1.18%), and for DII, to 2 out of 595 observations (0.34%).

Cross-regional variability was retained throughout the observation interval for the principal outcomes and resource indicators (Figure 1B). For physician visits per 1,000 population, a distinct trough was observed in 2020, followed by a return of the distribution to a higher range. For DII, the most pronounced upward shift in the median and upper quantiles was observed by 2024, whereas the distributions of the number of hospital organizations and ACO capacity changed far less markedly. At the national level, Figure 1C and Table 1 show two discontinuities in the telemedicine series: after 0.39–0.68 million consultations in 2018–2019, the volume increased to 7.00 million in 2020 and to 15.94 million in 2024, while the share of patient–physician consultations changed from 0.490 in 2018 to 0.919 in 2020 and then declined to 0.496 in 2024. Data-quality assessment did not reveal violations of the basic arithmetic invariants; however, the 2024 configuration limits direct comparison of proportional indicators across years. This compositional break indicates that the same nominal category of telemedicine consultations may not denote a temporally stable empirical object across the full observation period. The extended diagnostic layer, including comparison of the Russian Federation aggregate with the regional median for the principal indicators, verification of the proportional telemedicine indicator at the RF–year level, and the profile of extreme values across panel variables, is presented in Supplementary Data Sheet S3 and Supplementary File S1.

**Table 1:** National telemedicine exposure and data-quality flags, 2018–2024.

Year	Era	TM per 1,000	Patients per 1,000	Patient share	YoY % consults	YoY % patients
2018	Pre-COVID-19	2.6	1.3	0.490	—	—
2019	Pre-COVID-19	4.6	2.6	0.568	75.2	102.9
2020	COVID-19	47.4	43.6	0.919	931.7	1570.7
2021	COVID-19	58.6	51.6	0.880	23.3	18.1
2022	Post-COVID-19	52.1	46.0	0.883	−11.5	−11.3
2023	Post-COVID-19	55.8	47.9	0.857	6.9	3.8
2024	Post-COVID-19	109.1	54.1	0.496	95.1	13.0

The share of patient–physician consultations was calculated as the ratio of patient–physician consultations to the total number of telemedicine consultations. Annual changes were calculated relative to the preceding year; for 2018, they are undefined. The arithmetic consistency of the series is preserved in all years; however, the sharp decline in the share of patient–physician consultations in 2024 points to a possible shift in flow composition and/or accounting rules.

Subsequent coefficients should be interpreted in light of two underlying constraints: the selective missingness profile affecting the socioeconomic and tariff covariates, and the measurement instability of the national telemedicine signal.

### 3.2 Calibration of the digital signal: cross-regional structure versus within-region dynamics

**Figure 2:**
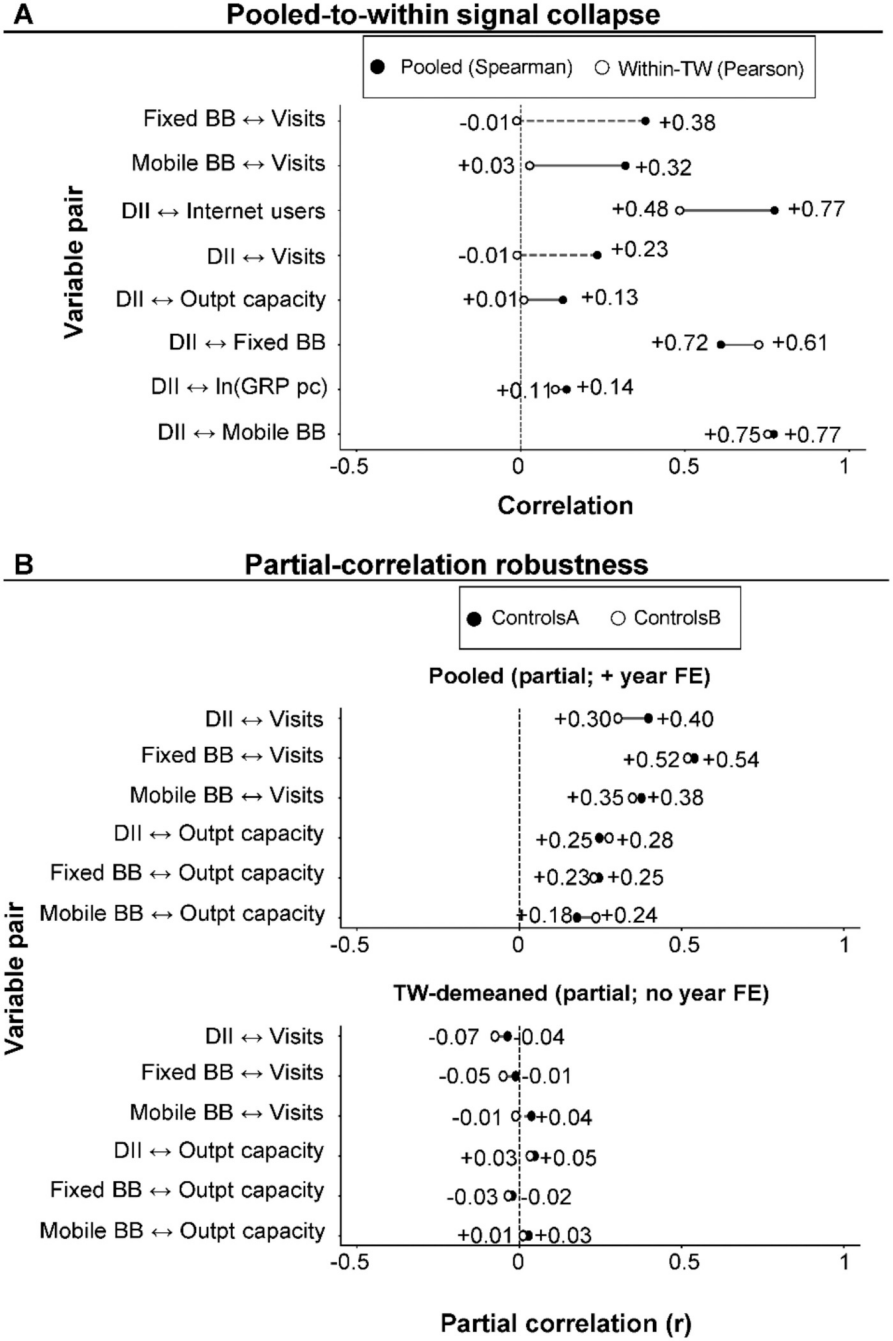
Calibration of the digital signal: transition from the pooled to the within-region specification and robustness to alternative control sets. **(A)** Comparison of pooled correlations and correlations after two-way centering for selected digital pairs. For the associations of digital indicators with physician visits and ACO capacity, the cross-regional associations attenuate substantially after removal of region and year means, whereas the associations of DII with components of the digital environment remain high. **(B)** Comparison of partial correlations under control set A and control set B in the pooled and within-region specifications. In the pooled setting, expansion of the control set shifts the estimates only moderately while preserving their sign and order of magnitude; in the within-region setting, partial correlations remain compressed toward the vicinity of zero. Note. The figure uses the following labels: DII, fixed BB, mobile BB, physician visits, and ACO capacity.

As shown in Table 2 and Figure 2A, the transition from the pooled specification to the within-region specification after two-way centering markedly attenuated the digital associations. For DII, the association with physician visits per 1,000 population changed from +0.258 in the pooled specification to −0.031 after two-way centering; for ACO capacity per 10,000 population, the corresponding estimates shifted from +0.087 to −0.003.

**Table 2:** Digital signal in the region–year panel: correlations in the pooled specification and after two-way centering.

Digital indicator (X)	Outcome (Y)	Pooled	Within (TW)	Partial pooled, ControlsB	Partial within (TW), ControlsB
DII	Visits per 1,000	+0.258	−0.031	+0.236	−0.041
DII	ACO capacity per 10,000	+0.087	−0.003	+0.153	+0.044
Fixed broadband (per 100)	Visits per 1,000	+0.424	−0.037	+0.497	−0.053
Fixed broadband (per 100)	ACO capacity per 10,000	+0.140	−0.055	+0.185	−0.062
Mobile broadband (per 100)	Visits per 1,000	+0.365	+0.018	+0.375	−0.009
Mobile broadband (per 100)	ACO capacity per 10,000	+0.250	+0.056	+0.166	+0.035
Internet subscription fee	Visits per 1,000	+0.071	−0.150	−0.239	−0.164
Internet subscription fee	ACO capacity per 10,000	−0.010	−0.024	−0.255	−0.010

Pooled correlations predominantly reflect cross-regional co-variation. Correlations after two-way centering were calculated after removal of region and year means and capture within-region deviations relative to those means. Partial correlations were estimated under control set B, which included the logarithm of GRP per capita, physician density, mid-level medical personnel density, and the number of hospital organizations per 100,000 population. This table is used to calibrate signal structure and does not substitute for regression analysis.

A similar profile was observed for the individual digital components. For fixed BB with physician visits as the outcome, the estimate shifted from +0.424 to −0.037; for mobile BB, it shifted from +0.365 to +0.018. For ACO capacity as the outcome, the corresponding transitions were +0.140 to −0.055 for fixed BB and +0.250 to +0.056 for mobile BB. For the Internet access tariff indicator, the association with physician visits remained weakly positive in the pooled specification (+0.071), but moved into a more distinctly negative range after two-way centering (−0.150), whereas for ACO capacity the estimates remained close to zero (−0.010 and −0.024). This indicates that, in the present dataset, the more pronounced digital signal was expressed in the cross-regional dimension, whereas within-region deviations over 2018–2024 generally yielded estimates clustered around zero. The extended diagnostic layer for this block is presented in Supplementary Data Sheet S2 and Supplementary File S2.

The same pattern was retained after partial control for the socioeconomic and resource-related contour. As shown in Figure 2B, in the pooled specification, the transition from control set A to control set B only moderately reduced the partial correlation of DII with physician visits per 1,000 population, from +0.449 to +0.236, and with ACO capacity, from +0.210 to +0.153. For fixed BB with physician visits as the outcome, the corresponding shift was +0.561 to +0.497; for mobile BB, it was +0.447 to +0.375. After two-way centering, those same associations remained near zero: for DII with physician visits, the estimate remained at −0.041 under both control sets; for ACO capacity, it changed only from +0.050 to +0.044; and for fixed BB with physician visits as the outcome, it shifted from −0.029 to −0.053.

The multicollinearity diagnostic contour remained moderate overall. In the specification with DII, the VIF values were low, amounting to 1.16 for DII, 1.92 for the logarithm of GRP per capita, and 2.22 for mid-level medical personnel density. In the digital block, higher values were observed for the share of households with Internet access (5.19) and the number of Internet users per 100 population (4.97), whereas for fixed BB and mobile BB they remained substantially lower. The condition number likewise remained within a non-alarming range: approximately 5.70 for the specification with disaggregated digital indicators and approximately 2.70 for the block including DII together with resource and socioeconomic variables. This indicates moderate, but not critical, interrelatedness among the predictors. Taken together, this pattern suggests that digital health indicators in the present setting should be interpreted primarily as markers of territorially structured context, rather than as direct proxies of short-term within-region expansion in ambulatory access.

The attenuation of digital associations after two-way centering indicates that the digital signal in this panel is structured to a substantial extent by interregional differences. This is consistent with an interpretation of the digital divide at the territorial level, but it does not establish whether telemedicine reduced or amplified access inequality at the patient level.

### 3.3 The national telemedicine signal: short-series sensitivity and measurement stability

In the RF–year block, DII, demographic series, and resource indicators were used as contextual system indicators through which the stability of co-movement between the national telemedicine series and different layers of the macro-environment was assessed. The most consistent profile in this contour was observed for DII: as shown in Table 3, in the baseline levels model, its co-movement with the telemedicine series remained positive and statistically supported [*β* = 0.0221; 95% confidence interval (CI) 0.0152–0.0289; *p* = 0.00043]. After introduction of a linear trend, the estimate retained a positive direction but moved into a borderline zone of statistical support (*β* = 0.0120; 95% CI −0.00069–0.0247; *p* = 0.058), while in first differences a similar profile was reproduced with greater interval uncertainty (*β* = 0.0113; 95% CI −0.00150–0.0242; *p* = 0.070).

**Table 3:** Signal sensitivity of national telemedicine co-movement with contextual system indicators, 2018–2024.

Contextual system indicator	M0: levels	M1: levels + linear trend	M3: first differences
DII	0.0221*** [0.0152; 0.0289]	0.0120* [−0.00069; 0.0247]	0.0113* [−0.00150; 0.0242]
Mortality rate per 1,000	0.0364 [−0.0440; 0.1169]	0.1215** [0.0163; 0.2267]	0.1007 [−0.0450; 0.2463]
Natural increase per 1,000	−0.0619* [−0.1244; 0.00066]	−0.1195** [−0.2265; −0.0126]	−0.0938 [−0.2361; 0.0485]
Birth rate per 1,000	−0.0254** [−0.0493; −0.00155]	0.0020 [−0.0194; 0.0234]	0.0069 [−0.0159; 0.0297]
Physicians per 10,000	0.0361** [0.00955; 0.0626]	−0.00428 [−0.0503; 0.0417]	−0.00591 [−0.0472; 0.0354]
Mid-level medical personnel per 10,000	−0.0424** [−0.0727; −0.0121]	0.00358 [−0.0493; 0.0565]	−0.00288 [−0.0484; 0.0426]

DII, demographic indicators, and resource indicators were used as contextual system indicators to assess the directionality and specification sensitivity of the national telemedicine signal. They were not interpreted as individual access outcomes or as evidence of a causal effect of telemedicine. M0 is the levels model; M1 is the levels model with a linear trend; M3 is the first-differences model, in which co-change between the contextual indicator and TM was estimated. Coefficients are presented as β [95% CI]. Given the short RF–year series (T = 7), the estimates should be read as indicators of signal directionality and sensitivity to specification.

* *p* < 0.10.

** *p* < 0.05.

*** *p* < 0.01.

**Figure 3:**
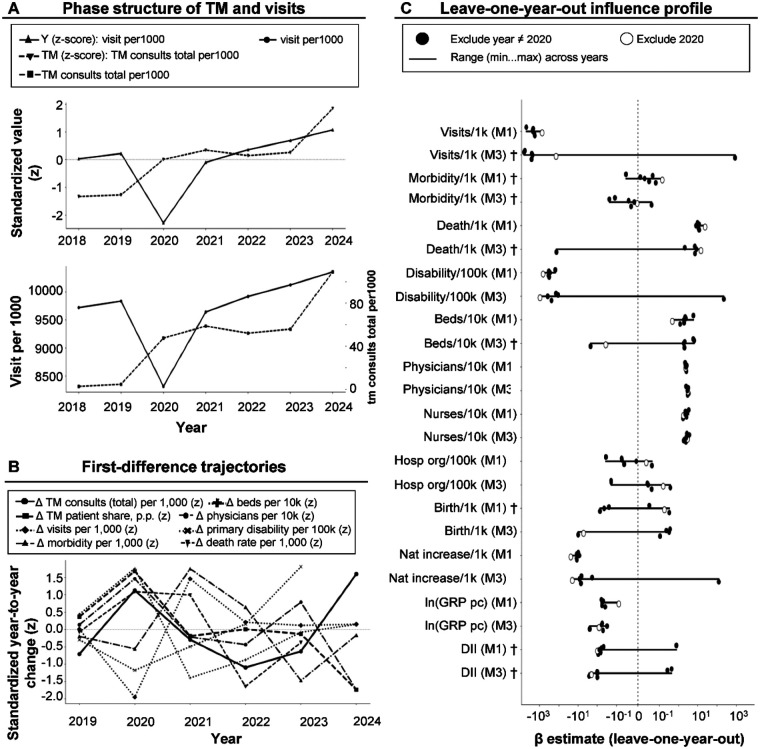
National telemedicine signal: short-series sensitivity and measurement stability. **(A)** Superimposed trajectories of TM and physician visits per 1,000 population at the RF–year level: the upper panel shows standardised series, and the lower panel shows levels on the original scale. **(B)** Standardised YoY changes in TM and selected contextual system indicators, showing the absence of a stable pattern of parallel movement. **(C)** Profile of sequential leave-one-year-out sensitivity in β estimates under specifications M1 and M3: white points correspond to exclusion of 2020, black points to exclusion of any other year, and horizontal segments indicate the range of estimates across all runs.

For contextual demographic indicators, the signal was less stable and appeared mainly in specification M1: for the mortality rate, the estimate was *β* = 0.1215 (95% CI 0.0163–0.2267; *p* = 0.0349), and for natural increase, *β* = −0.1195 (95% CI −0.2265–−0.0126; *p* = 0.0379). For the birth rate and workforce indicators, statistical support was largely confined to the baseline levels model M0 and weakened after trend adjustment or transition to first differences. At the same time, Figure 3A indicates phase heterogeneity within the national telemedicine series itself: the sharp increase in 2020, the elevated plateau in the subsequent years, and the renewed acceleration in 2024 unfolded against a non-synchronous trajectory of physician visits per 1,000 population. The extended map of signal directionality and specification sensitivity is presented in Supplementary Data Sheet S5; additional short-series diagnostic materials are provided in Supplementary File S3.

The transition from M0 to M1 and M3 was accompanied by attenuation of some signals observed in the levels models after removal of the common temporal component. As shown in Figure 3B, in first differences, the main annual changes in TM were concentrated in 2020 and 2024, whereas changes in the contextual indicators were distributed across years in a less concordant manner and, given T = 7, did not form a stable pattern of parallel movement. In the sequential leave-one-year-out procedure (Figure 3C), the largest shift in *β* across many rows occurred when 2020 was excluded. For DII, the mortality rate, and natural increase, the direction of the signal was preserved more frequently than for physician visits, indicating greater sensitivity of the latter series to the exclusion of individual years.

This profile should not be read as an assessment of telemedicine effectiveness. Rather, it indicates that macro-level telemedicine counters become an unstable interpretive instrument when the legal regime, the composition of the service flow, and measurement conditions change over time. The resulting configuration does not support reading the national telemedicine series as a stable and unambiguous indicator of expanded realised contact. It is more consistent with regime-sensitive redistribution of modalities and channels within an already existing flow of healthcare utilisation, under continuing measurement constraints.

### 3.4 Regional structural panel: fixed-effects estimates, effective coverage, and substantive coefficient profiles

The resource-based TWFE model for physician visits per 1,000 population used 595 observations across 85 territories over 2018–2024. Specification S3, which incorporated DII, resource indicators, and the socioeconomic block, used 510 observations across the same 85 territories over 2018–2023; the digital-components specification S4 used 473 observations across 79 territories over 2018–2023; and the FD model S8 used 425 observations across 85 territories over 2019–2023. For ACO capacity per 10,000 population, the corresponding figures were 588, 504, 467, and 420 observations.

**Figure 4:**
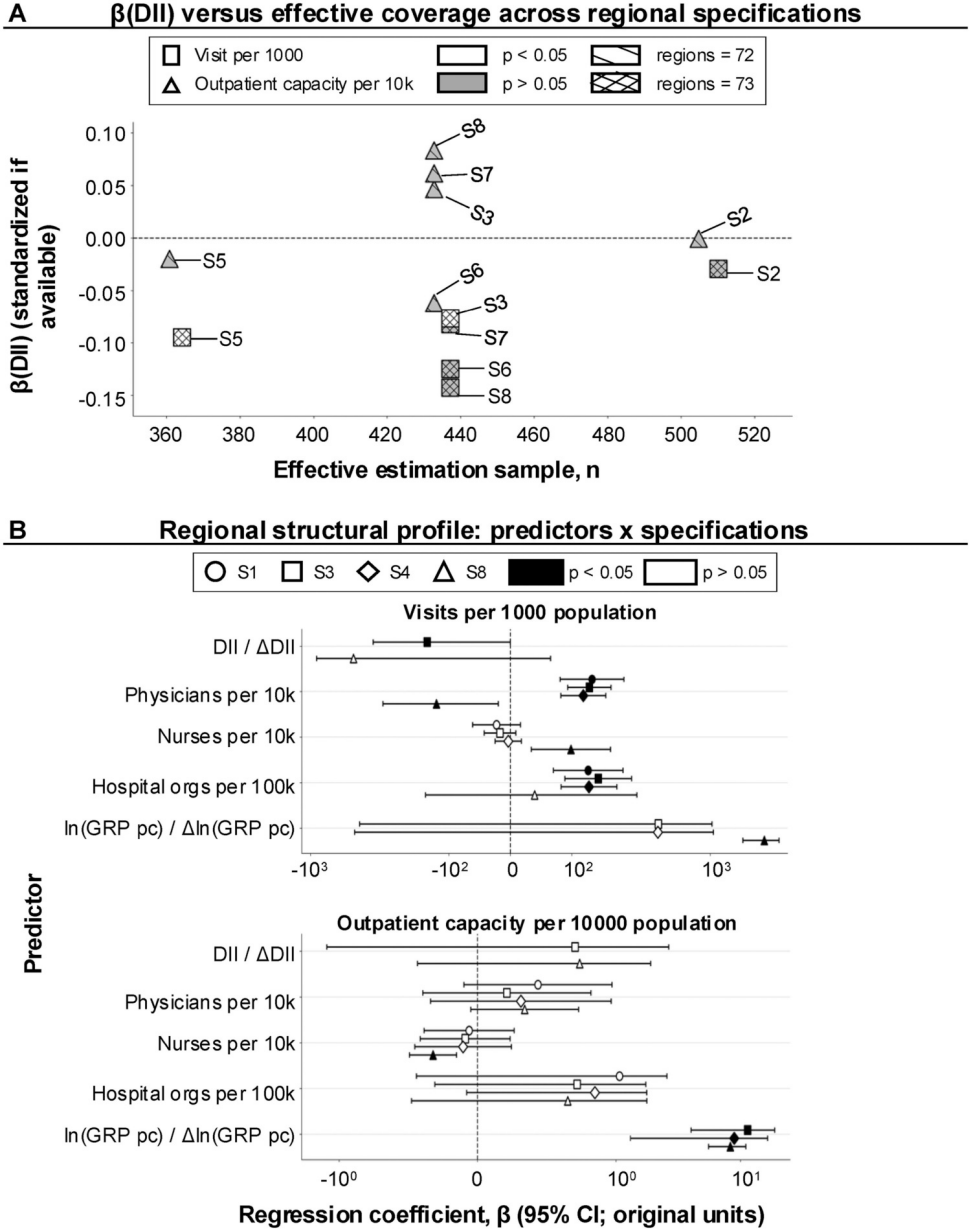
Regional structural contour: effective coverage and coefficient profiles. **(A)** Estimates of *β*(DII) in regional specifications containing DII as a function of effective coverage. **(B)** Coefficient profiles (β, 95% CI) for S1, S3, S4, and S8 across the two outcomes: the upper subpanel shows physician visits, and the lower subpanel shows ACO capacity. The vertical dashed line corresponds to *β* = 0.

Against this effective-coverage profile, Figure 4A and Supplementary Data Sheet S4 show that, in regional specifications containing DII, the digital index exhibited different profiles across the two outcomes. In the physician-visit models, the DII coefficient retained a negative direction: in specification S3, the estimate remained statistically uncertain [*β* = −80.19 (−237.64; 77.26)], whereas in the FD contour S8 it moved into a formally supported negative range [*β* = −442.38 (−878.59; −6.17)]. For ACO capacity, the DII estimates were concentrated near zero: 0.790 [−0.878; 2.457] in S3 and 0.701 [−0.317; 1.718] in S8. This pattern indicates that the digital index in the region–year panel retained a specification-sensitive profile and did not form a stable positive signal with respect to the expansion of ambulatory contact.

The substantive structure of the coefficients also differed between physician visits and ACO capacity (Table 4; Figure 4B). In the physician-visit models, the most coherent signal passed through the resource contour: coefficients for physician density in the main specifications remained positive and statistically supported, ranging from 110.49 to 199.90, while estimates for the number of hospital organizations ranged from 129.27 to 136.40. Against this background, the negative direction of DII indicates that observed contact intensity was more strongly associated with the resource endowment of ambulatory care than with the digital context itself. For ACO capacity, the configuration was different: DII remained statistically uncertain, resource coefficients were less pronounced, and the most orderly positive gradient passed through ln(GRP per capita), with estimates of 9.478 in S3, 12.389 in S4, and 7.876 in S8.

**Table 4:** Regional structural models: effective coverage and selected coefficient estimates by specification.

Predictor/coverage	S1 TWFE resources	S3 TWFE DII + resources + socio	S4 TWFE digital components	S8 FD
Panel A. Visits per 1,000 population				
N obs/regions/years	595/85/2018–2024	510/85/2018–2023	473/79/2018–2023	425/85/2019–2023
DII/ΔDII	—	−80.19 [−237.64; 77.26]	—	−442.38** [−878.59; −6.17]
Physician density per 10,000	189.12*** [101.09; 277.14]	199.90*** [104.96; 294.83]	110.49*** [70.93; 150.06]	−128.38*** [−212.46; −44.31]
Hospital organizations per 100,000	129.27*** [71.81; 186.73]	136.40*** [82.16; 190.63]	135.49*** [87.85; 183.12]	59.36 [−122.41; 241.12]
ln(GRP pc)/Δln(GRP pc)	—	460.62 [−110.35; 1031.59]	346.84 [−282.07; 975.75]	3378.09*** [2579.88; 4176.30]
Panel B. ACO capacity per 10,000 population				
N obs/regions/years	588/84/2018–2024	504/84/2018–2023	467/78/2018–2023	420/84/2019–2023
DII/ΔDII	—	0.790 [−0.878; 2.457]	—	0.701 [−0.317; 1.718]
Physician density per 10,000	0.021 [−0.493; 0.534]	−0.241 [−0.735; 0.253]	0.094 [−0.531; 0.719]	0.248 [−0.049; 0.545]
Hospital organizations per 100,000	1.156 [−0.242; 2.554]	0.774 [−0.185; 1.733]	0.767 [−0.181; 1.715]	0.706 [−0.353; 1.765]
ln(GRP pc)/Δln(GRP pc)	—	9.478*** [3.507; 15.448]	12.389*** [6.425; 18.353]	7.876*** [5.734; 10.019]

S1 denotes the resource model with TWFE; S3, the model with DII and the resource and socioeconomic blocks; S4, the model with disaggregated digital components; and S8, the FD model. Cells report coefficients β [95% CI]. The first row of each panel shows the effective coverage of the corresponding specification. For the FD model S8, the first row reports the effective number of observations, regions, and years used for estimation after transition to FD. An empty cell indicates that the predictor was not included in the model.

The symbols indicate statistical significance levels.

*p < 0.10.

**p < 0.05.

*** *p* < 0.01.

Accordingly, in the regional structural panel, physician visits exhibited a more coherent resource profile under a persistently negative DII direction, whereas for ACO capacity the most orderly positive gradient ran through the economic rather than the digital contour.

### 3.5 Robustness checks and the boundaries of interpretation

**Figure 5:**
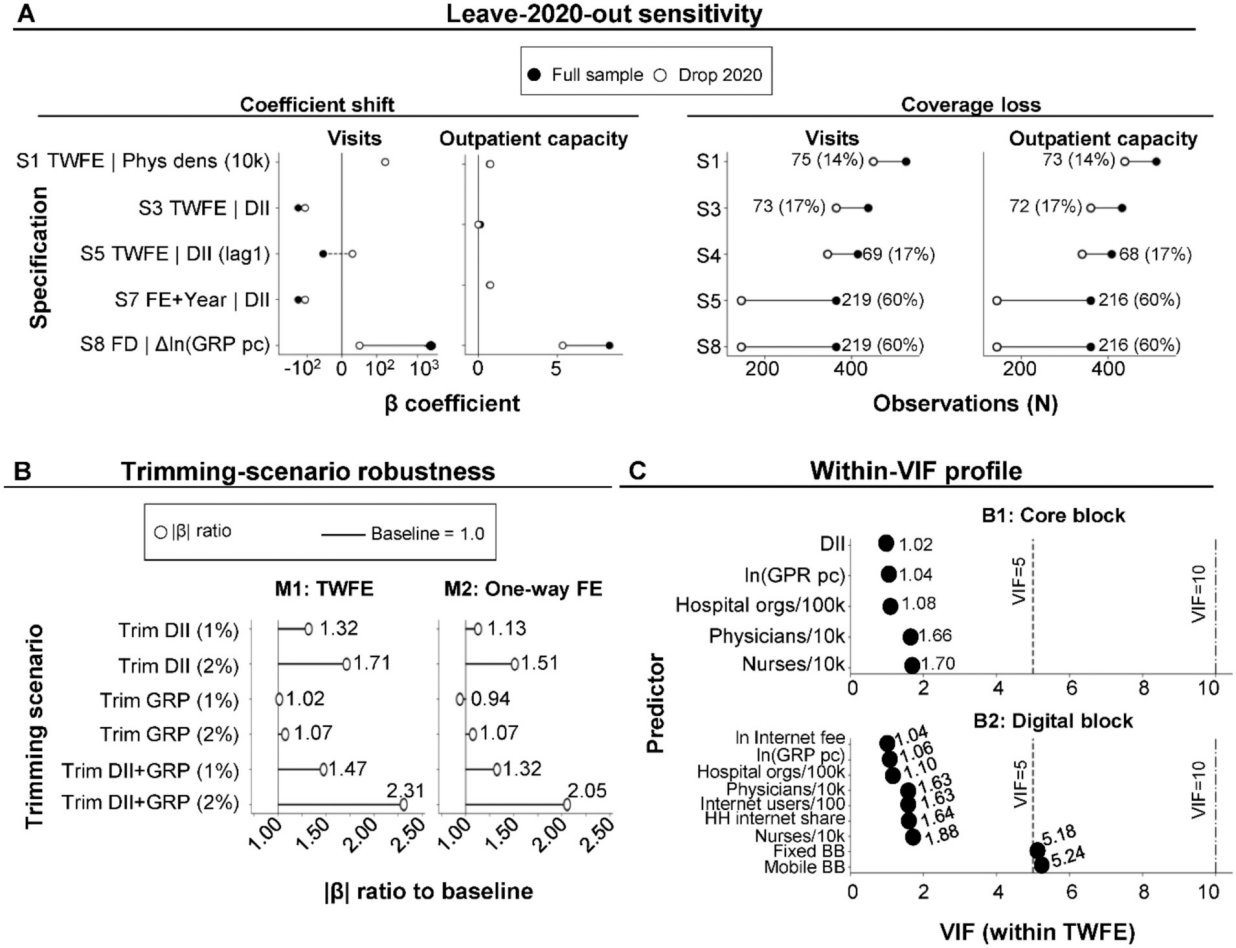
Robustness checks and the boundaries of interpretation. **(A)** Left: comparison of β coefficients in the full sample and after exclusion of 2020 for selected regional specifications; right: the corresponding loss in the number of observations. Filled points correspond to the full sample, open points to the sample excluding 2020; the dashed connector denotes a sign change. **(B)** The ratio |β scenario|/|β baseline model| in tail-trimming scenarios for physician visits in specifications with TWFE and one-way fixed effects; the vertical line corresponds to the baseline level = 1.0. **(C)** The within-region VIF profile for the core model and the digital block; the dashed and dash-dotted vertical lines correspond to the thresholds VIF = 5 and VIF = 10.

When 2020 was excluded, the negative direction of the association between DII and physician visits was preserved in the levels-based contours, although sensitivity increased in the more demanding specifications (Figure 5A; Table 5). For physician visits, the DII estimate in S3 shifted from −80.19 to −51.18, whereas in the lagged specification S5 the negative coefficient retained its sign and moved into a more deeply negative range (−110.83 to −446.99). In the FD model S8, the coefficient on Δln(GRP per capita) also declined sharply, from 3378.09 to 228.51. For ACO capacity, the most pronounced shifts were concentrated in the lagged contour: the coefficient on lagged DII changed from 0.076 to −1.208, whereas in the FD model the coefficient on Δln(GRP per capita) remained close to its original level (7.876–7.480). Direct interpretation of these differences is constrained by the fact that effective-coverage losses remained moderate in S1–S4, whereas in S5 and S8 they became substantially more pronounced.

**Table 5:** Robustness profile of the DII effect in the region–year panel.

Panel A. Leave-one-region-out stability						
Outcome	Specification	β (full sample)	β range under LOO	Max |Δβ|	Sign flip	Most influential region
Visits per 1,000 population	TWFE core	−80.19	−125.25–−53.69	45.06	No	Moscow
Visits per 1,000 population	Region FE + year dummies	−80.19	−125.25–−53.69	45.06	No	Moscow
Outpatient capacity per 10,000 population	TWFE core	0.79	0.48–1.56	0.77	No	Kirov Oblast
Outpatient capacity per 10,000 population	Region FE + year dummies	0.79	0.48–1.56	0.77	No	Kirov Oblast

Panel B. Scenario sensitivity for the DII effect on visits (TWFE core)					
Scenario	β(DII)	*p* (cluster: region)	|β|/|β₀|	N obs	N regions
Exclude capital cities	−80.19	0.318	1.000	510	85
Trim DII (2% tails)	−119.49	0.434	1.490	486	84
Trim DII + GRP per capita (2% tails)	−218.93	0.154	2.730	462	81
Drop year 2020	−51.18	0.543	0.638	425	85
Drop years 2020–2021	−57.29	0.536	0.714	340	85

Panel C. Sensitivity to variance estimation					
Outcome	Specification	β(DII)	*p* (region cluster)	*p* (HC3)	*p* (year cluster)
Visits per 1,000 population	TWFE core	−80.19	0.318	0.275	0.284
Visits per 1,000 population	Region FE + year dummies	−80.19	0.321	0.280	0.286
Outpatient capacity per 10,000 population	TWFE core	0.79	0.353	0.344	0.330
Outpatient capacity per 10,000 population	Region FE + year dummies	0.79	0.356	0.350	0.333

Panel D. Within-VIF summary			
Block	Variable	VIF	N obs
Core (DII + resources + socioeconomics)	DII	1.010	510
Core (DII + resources + socioeconomics)	ln(GRP per capita)	1.014	510
Core (DII + resources + socioeconomics)	Nurses per 10,000	1.734	510
Digital block	Mobile broadband per 100	4.088	473
Digital block	Fixed broadband per 100	4.055	473

TWFE denotes the model with two-way fixed effects; HC3 denotes the heteroskedasticity-consistent covariance estimator. Panel A summarizes the results of re-estimating the DII coefficient under a sequential leave-one-region-out scheme. Panel B presents the scenario analysis only for the baseline TWFE model for physician visits; β₀ denotes the baseline DII estimate in the main specification. Panel C compares statistical inference under alternative variance-estimation schemes with the point estimate held constant. Panel D reports VIF values after the within-region transformation.

For physician visits, after exclusion of 2020, effective coverage declined from 595 to 510 observations in S1, from 510 to 425 in S3, and from 473 to 394 in S4, corresponding to losses of 14.3–16.7%; in S5 and S8, however, it fell to 170 observations, corresponding to a loss of approximately 60.0%. For ACO capacity, the pattern was virtually the same: 588–504 in S1, 504–420 in S3, 467–389 in S4, and 420–168 in S5 and S8.

The reduction in effective coverage imposes narrower conditions for reading the more demanding specifications: lagged models and FD specifications function as a sensitive robustness-testing contour, while their interpretive weight is conditioned by the lower completeness of available observations compared with the baseline TWFE models.

Under tail-trimming scenarios, the negative DII signal for physician visits was preserved, although the magnitude of the estimate changed unevenly (Figure 5B; Table 5B). Additional robustness checks, sensitivity analyses for standard errors, and diagnostics of the within-region correlation structure of the predictors are presented in Supplementary Data Sheet S6 and Supplementary File S4. In the baseline TWFE specification, trimming DII alone increased the absolute magnitude of the coefficient to 1.49 times its original level, whereas joint trimming of DII and GRP per capita increased it to 2.73. By contrast, exclusion of 2020 and exclusion of 2020–2021 attenuated the absolute magnitude of the estimate to 0.64 and 0.71 of the baseline value, respectively. This configuration indicates that the magnitude of the signal was more sensitive to the tails of the DII and GRP distributions than to the exclusion of individual COVID-19 years, while preserving its overall negative direction.

The boundary of interpretation was further refined through the uncertainty-estimation scheme and the profile of within-region collinearity (Figure 5C; Tables 5C,D). For the “physician visits × DII” pairing, the *p*-value was 0.318 under clustering by region, 0.275 under HC3, and 0.284 under clustering by year; for ACO capacity, the corresponding values were 0.353, 0.344, and 0.330. At the same time, the leave-one-region-out profile remained sign-stable: neither for physician visits nor for ACO capacity was a sign inversion observed. Within-region collinearity in the core model remained low: VIF was 1.010 for DII, 1.014 for ln(GRP per capita), and 1.734 for mid-level medical personnel density. In the digital block, the values were higher but remained within a moderate range: 4.088 for mobile BB and 4.055 for fixed BB (Table 5D).

## 4 Discussion

### 4.1 Territorial digital stratification and attenuation of within-region signals

In the region–year panel, the most pronounced association between the digital environment and ambulatory indicators was expressed in the cross-regional dimension: after two-way centering, the digital associations contracted markedly, while the within-region estimates shifted toward the null range. This profile indicates that the initial pooled signal primarily reflected stable territorial stratification. Within this configuration, territorial digital endowment is linked to the resource organisation of the ambulatory sector; the conditions of remote entry are formed within an infrastructural environment already anchored at the regional level. This interpretation is consistent with evidence on the territorial heterogeneity of the remote channel: telemedicine use is associated with rural territorial profiles and the quality of BB access; Medicare data further specify the infrastructural side of this profile through the association between BB infrastructure capacity and the level of telemedicine use (10, 11). For the short 2018–2024 panel, such attenuation is methodologically expected, since digital infrastructure changes inertially, while the transition from the technical attainability of the remote channel to realised physician contact requires organisational alignment and stable service practices.

In the Russian case, this result acquires an additional institutional dimension. DII reflects the region's position within the state-organised digital healthcare contour associated with the trajectory of EGISZ and the 2019–2024 federal project: medical information systems became part of the infrastructure for interaction with EGISZ, while remote services were incorporated into a governed digital architecture (26, 35). At the same time, assessments of digital maturity across Russian Federation regions and healthcare organisations show uneven depth of digital-solution implementation; the organisational level requires separate attention because the differences extend to infrastructural components and workforce capacity to sustain the digital space of a healthcare organisation (36, 37). The attenuation of within-region estimates should therefore be read as indicating that, in this panel, DII functions primarily as a territorial implementation context, where the significance of digitalisation is expressed through stable interregional differences in implementation and only a limited role of short-term within-region dynamics. Its relationship to realised ambulatory contact operates through the factual traversability of the remote channel: EGISZ and regional medical information systems provide the institutional infrastructure of digital entry, while the stability of the remote care-delivery channel within the workflow is determined by the organisational capacity of healthcare organisations (38).

### 4.2 Limits of the equalisation interpretation of telemedicine growth

The findings of the present study limit a direct reading of TM growth as evidence of access equalisation. In the region–year panel, DII functioned primarily as a characteristic of the territorial digital context: after two-way centering, the digital associations contracted, while the association with physician visits did not form a stable positive profile. The discussion of the distributional effect of telemedicine should therefore shift from the mere existence of the remote care-delivery channel to the conditions under which the digital scenario is practically traversable. Evidence on telephone and audio-only formats shows that a registered remote contact may serve as an entry point for vulnerable patients while preserving differences in the attainability of more demanding digital modalities (13, 14). The organisational layer sets a further boundary of interpretation: the conversion of territorial infrastructural readiness into a stable service process depends on the institution's capacity to embed remote contact into the workflow and on its level of digital maturity (19, 20). Patient trust and workforce preparedness further determine whether telemedicine contact is sustained in practical use, making any distributional inference dependent on the conditions of implementation (22, 39).

In the Russian material, this boundary acquires a pronounced patient-side dimension. Evidence on digital access to healthcare in Russia shows that the use of digital tools for managing medical appointments and health information remains socially uneven: almost half of respondents did not use such tools, and differences were associated with age, education, Internet activity, and preferred access device (40). Data on population engagement in healthcare digitalisation further show that the perceived reduction of time and financial costs remains dependent on digital skills, trust in the quality of care, and confidence in the security of personal data (41). The digital divide in the Russian context should therefore be understood as differences in the patient's practical capacity to enter the remote care-delivery channel and to keep the registered contact within an actually traversed pathway.

Within this framework, TM volume should be read as registered service activity: its distributional meaning is formed through modality structure and organisational and user-side traversability of the pathway. The contact counter alone is insufficient for inferring access equalisation, since growth in registered remote contacts may reflect expansion of the digital contour or a change in the composition of the service flow. Any inference about the realisation of access requires assessment of the contact's position within the patient pathway and its relationship to care actually received.

### 4.3 Outcome heterogeneity: contact intensity and structural capacity of the ambulatory sector

The heterogeneity of the two regional outcomes should be treated as a substantive finding, since physician visits per 1,000 population and ACO capacity per 10,000 population belong to different layers of the ambulatory care contour. The former indicator captures the aggregated intensity of realised contact, which is consistent with evidence on telemedicine as a mechanism that modifies contact burden and subsequent healthcare utilisation (6). The latter indicator describes the more inertial organisational capacity of the ambulatory network; therefore, its relationship with the digital index requires a more structural reading and is closer to the stable socioeconomic profile of the region.

The present findings fit within a broader logic of primary care: expanded availability of the remote channel may change the structure of contacts without a comparable increase in the overall level of utilisation, making the RCI proxy closer to the current routing of patient flow (8). Healthcare utilisation and unmet need refer to different dimensions of access; accordingly, contact activity should not substitute for the question of the completed patient pathway (31). Studies of primary care further show that the remote format reconfigures the modality of service delivery and subsequent pathway transitions, while the SCA proxy captures a more slowly changing organisational layer of the ambulatory sector (42, 43). Within this framework, the RCI proxy and the SCA proxy should be interpreted as interrelated dimensions of the regional ambulatory care contour, each describing its own layer of association between the digital context and ambulatory care.

### 4.4 Governing the measurement of the national telemedicine signal

In the present manuscript, the RF–year series functions as an audit of the interpretability of the telemedicine counter: the results show that the association between the telemedicine series and contextual indicators weakened after the introduction of temporal structure, while 2024 produced a compositional break in which growth in total volume was accompanied by a sharp decline in the share of patient–physician consultations. In the Russian material, this finding has an administrative-digital meaning, because the national telemedicine signal is formed within a state-organised digital healthcare contour associated with EGISZ and the unified digital contour; information exchange between healthcare organisations forms part of this architecture of observation (26, 35). Form No. 30 further positions telemedicine technologies as an object of official statistical reporting: the overall consultation flow is administratively recorded, while ambulatory telemedicine care and remote monitoring constitute separate segments of registered remote service delivery (44). The RF–year telemedicine series should therefore be read as an administratively produced measurement object. Its relationship to access depends on the stability of the counted category; modality structure and the composition of the registered flow define the next level at which the interpretability of the series must be assessed.

This dependence is reinforced by the legal and tariff layers of Russian telemedicine. The development of experimental legal regimes after 2020 reflects the continuing calibration of permissible remote care-delivery formats (45), while Russian data on telemedicine reporting show the sensitivity of registered volume to payment rules and regional tariff configuration (27, 30). International literature on telehealth measurement supports this reading, because the assessment of remote service delivery requires purpose-specific data preparation aligned with the particular managerial task (4). Studies of electronic records show that modality classification depends on record elements and billing capture; the literature on administrative data quality further emphasises the importance of coding comparability for the cross-period comparability of monitoring indicators (32, 33). In the Russian system, this risk takes an institutional form in the context of the modernisation of medical statistics, where the transition to primary data raises the requirements for the reliability of the reporting contour (46). The national telemedicine signal is therefore informative for monitoring phase changes and compositional shifts; any inference about access expansion requires documentation of reporting definitions and assessment of the stability of the counted object over time.

### 4.5 Implications for monitoring and interpretation

The practical implication of the present findings is a shift from a counter of remote consultations to a pathway-based architecture of telemedicine monitoring. Within such an architecture, volume retains value as a signal of scaling, while its interpretation is determined by which segment of the pathway becomes observable and which form of pathway loss can be distinguished from administrative recording through the registered contact. The results of the region–year panel show that the digital signal is predominantly embedded in territorial stratification; monitoring should therefore begin by characterising the territorial digital context as the infrastructural-resource environment of remote entry. The comparison of outcomes specifies the next level of interpretation: contact intensity and the structural capacity of the ambulatory sector describe different layers of the ambulatory contour, so telemedicine monitoring should link the registered contact to a specific patient pathway segment. The RF–year block further shows that the telemedicine series depends on the composition of the registered flow; at this level, modality structure constrains the admissible inference about realised contact and shifts the monitoring architecture into the domain of measurement governance.

For the Russian material, this sequence requires verification of reporting definitions and of the stability of the counted object, because registered telemedicine volume is formed within a state-organised digital healthcare contour and an official statistical regime. From the perspective of digital equity, monitoring should capture the fact of remote interaction together with those segments of the digital scenario in which the patient encounters entry friction and technical dependence, including the need for assisted navigation (47). TM volume therefore acquires interpretive value when the registered contact is related to the traversability of the patient pathway; the regional implementation context and the composition of the registered flow define the measurement boundary of such inference. A stable monitoring framework requires documented modality classification and reporting definitions that remain comparable over time, so that the factual scaling of the remote care-delivery channel can be distinguished from changes in the registered flow.

## 5 Limitations

The limitations of the present study define the level of admissible inference: the results should be read as aggregated regional and system-level signals. The observational ecological design, based on annual data for the constituent entities of the Russian Federation, does not allow associations to be transferred to the level of individual patients or completed episodes of care; the specifications used reduced the risk of overinterpretation while leaving room for residual confounding and endogeneity in telemedicine implementation. The national telemedicine series was available only at the RF–year level and was treated as an independent measurement signal outside the regional models; regional differences in registration procedures may have affected the comparability of official indicators. The 2018–2024 period remains short and includes phase discontinuities: 2020 reflects pandemic-related scaling of remote formats, while 2024 marks a compositional boundary in the series, since growth in total volume was accompanied by a sharp change in the share of patient–physician consultations. Selective missingness reduced the effective coverage of saturated specifications, especially in lagged models and FD specifications. The composite DII characterised the infrastructural and population-level digital context; platform quality and workforce readiness remained outside its measurement domain, as did the patient's ability to complete the remote scenario. Further validation should focus on the organisational mechanisms of digital adoption and the engagement of healthcare workers, with separate attention to patient losses at different stages of the remote pathway.

## 6 Conclusions

The findings do not support interpreting telemedicine growth as an autonomous indicator of access equalisation across regions of the Russian Federation. In the region–year panel, the digital signal appeared primarily as a territorially structured context, whereas the short-term within-region association with expanded ambulatory contact remained weak. Physician visits per 1,000 population and ACO capacity per 10,000 population described different layers of the ambulatory care contour: the former was closer to current contact activity, while the latter reflected the more inertial organisational capacity of the sector. The national telemedicine series remained sensitive to phase shifts and changes in the composition of the registered flow, which limits its use as a stable indicator of access expansion. TM volume should therefore be treated as a bounded monitoring signal whose interpretation depends on the observed segment of the patient pathway, the modality structure of contact, and the comparability of reporting definitions.

Further research should connect regional statistics with individual-level data on patient-pathway progression: from waiting and appointment confirmation to realised contact, and then to possible modality switching and episode completion. Such a design would allow a more precise distinction between redistribution of service channels and factual improvement in pathway traversability. A separate direction concerns the organisational layer of implementation. Panel data should be complemented by qualitative or mixed-methods studies of healthcare worker engagement as manifested in workflow adaptation and the routine use of telemedicine platforms, with specific attention to the role of managerial and technical participants in shaping the digital process. Within this framework, telemedicine should be evaluated as a pathway-based service configuration in which access depends on the system's verifiable capacity to guide the patient from digital entry to a completed episode of care.

## Data Availability

The original contributions presented in the study are included in the article/Supplementary Material, further inquiries can be directed to the corresponding author.
