# Supplementary material for: Digital infrastructure and proxies of ambulatory care access in Russia, 2018–2024: a regional panel study with a national telemedicine signal analysis
Source: Front Digit Health. 2026 Jun 23;8:1856577. doi: 10.3389/fdgth.2026.1856577 (PMC13338863; doi:10.3389/fdgth.2026.1856577)
Supplement: Supplementary file 1 [file Supplementaryfile1.pdf]

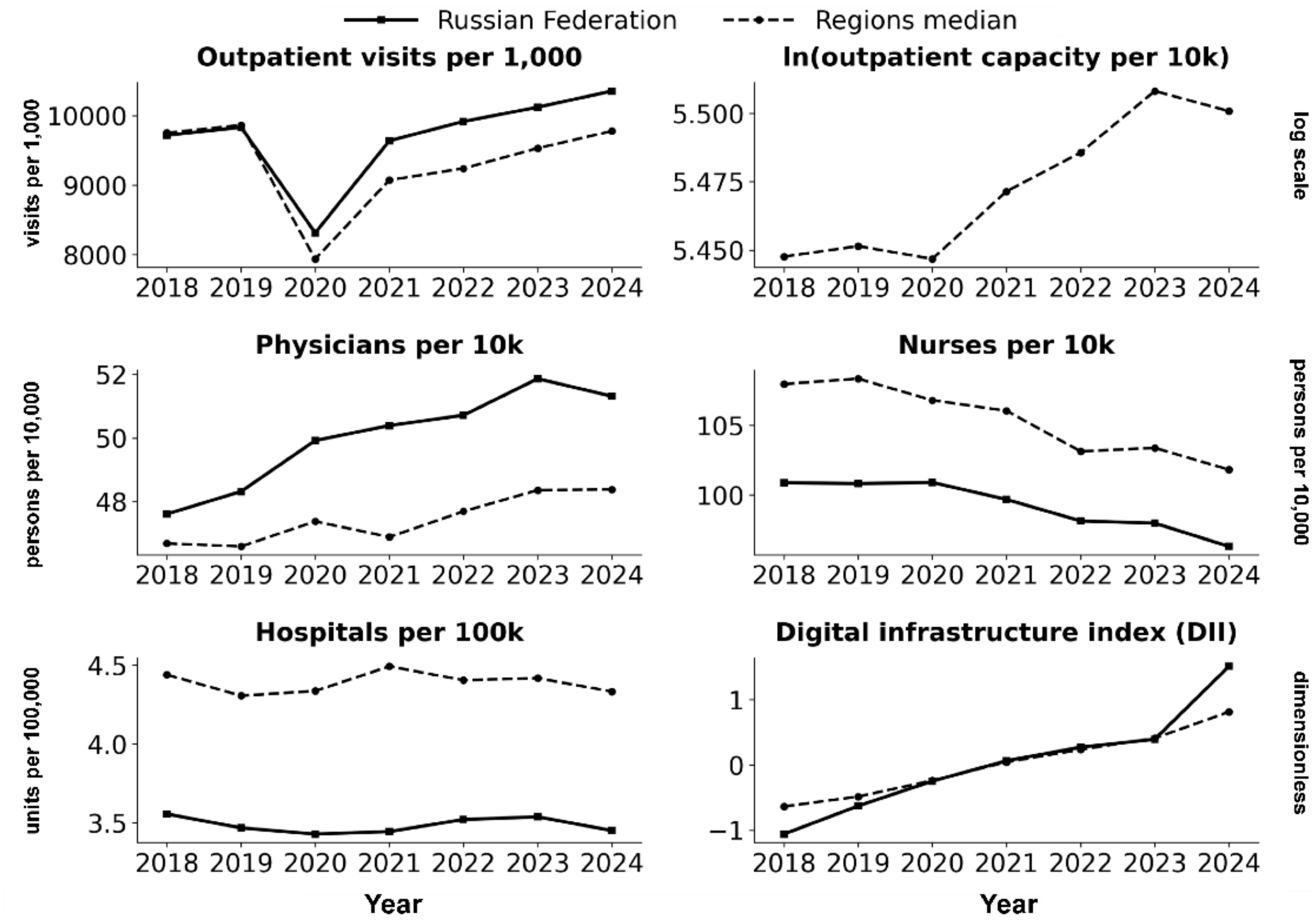

Figure S1. Trends in the Russian Federation aggregate and the regional median across indicators, 2018–2024. The figure juxtaposes the trajectories of the aggregate value for the Russian Federation and the median of regional values for six indicators over 2018–2024; the solid line denotes the RF aggregate, the dashed line the regional median, and the figure itself is constructed from annual summary tables, without recourse to raw microdata. For visits per 1,000, both series display a pronounced trough in 2020 followed by recovery, with the RF aggregate lying above the regional median after

2020. For ln(outpatient capacity per 10k), the two lines almost completely overlap throughout the period, and the indicator follows a smooth upward trajectory. For physicians per 10k, both trajectories rise, while the RF line remains consistently above the regional median, and a gap between them is still evident by 2024. For nurses per 10k, the regional median remains above the RF aggregate throughout the entire interval; both series show a moderate downward movement. For hospitals per 100k, the RF line remains stably below the regional median and changes only marginally, whereas the regional median stays higher throughout the observation period. For DII, both series exhibit an upward trend; however, in 2024 the aggregate RF value increases markedly faster than the regional median, with the result that the divergence between the two lines reaches its maximum precisely at the terminal point of the series. Taken together, the figure documents an overall concordance in the shape of movement between the RF aggregate and the regional median, while preserving differences in level and amplitude for particular indicators, most notably visits, workforce metrics, and DII.

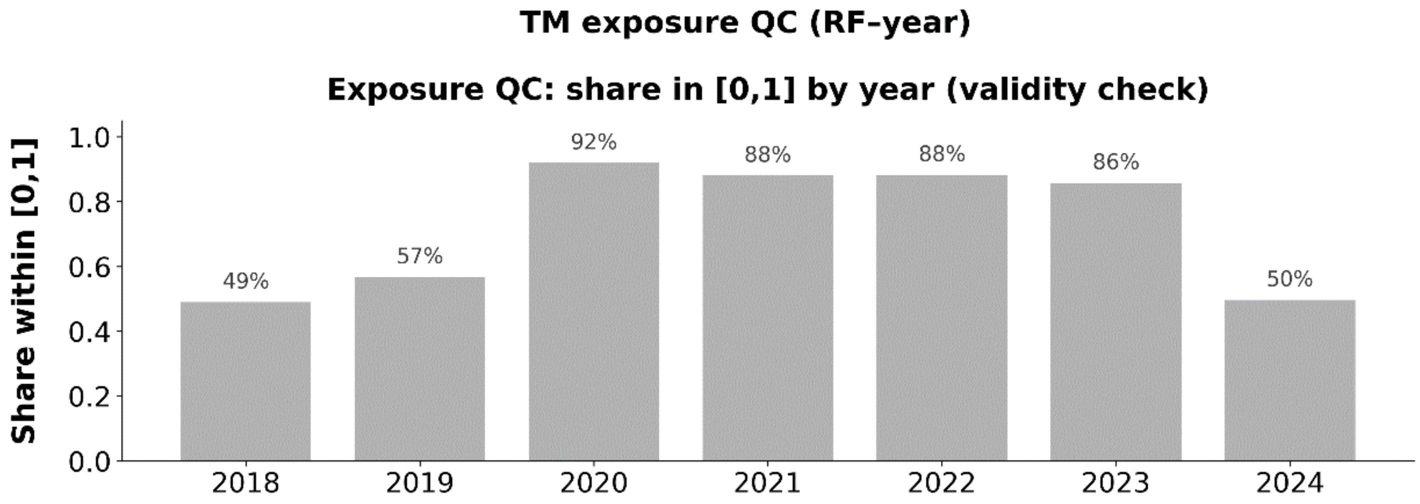

Figure S2. QC of telemedicine exposure at the RF–year level, 2018–2024. The figure presents a quality-control assessment of the share-based telemedicine exposure indicator at the RF–year level by verifying, for each observation year, that it remains within the admissible interval [0, 1]. In every year, the indicator retains formal range-validity; however, its temporal profile remains heterogeneous: the value is 49% in 2018, 57% in 2019, 92% in 2020, 88% in 2021, 88% in 2022, 86% in 2023, and 50% in

2024. Accordingly, during 2020–2023 the share remains within a higher corridor of 86–92%, whereas the initial years and 2024 form distinct points with markedly lower values. Considered together with the QC checks applied to the series, this indicates that the indicator preserves its basic internal arithmetic consistency: the patient share remains within [0, 1], deriving the share from its components yields no meaningful discrepancy, and the condition “patient consultations ≤ total consultations” holds in every year. At the same time, the figure makes clear that the internal consistency of the series does not eliminate the temporal heterogeneity of the share profile itself: after rising to 0.919 in 2020, the indicator remains at 0.857–0.883 in 2021–2023, before declining to 0.496 in 2024.

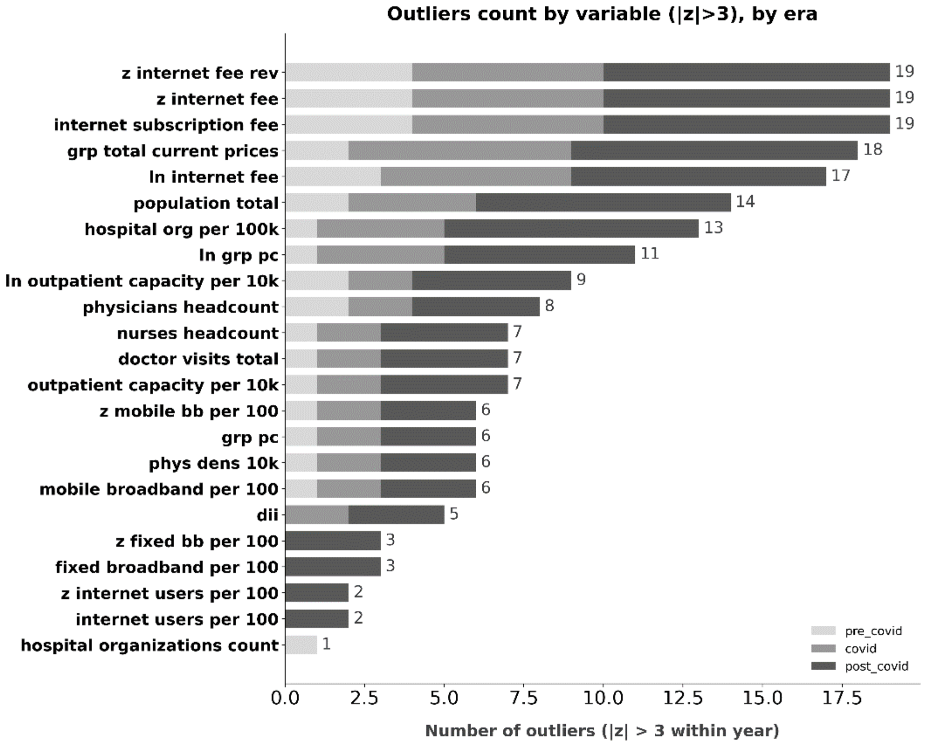

Figure S3. Number of outliers by variable (|z| > 3), stratified by observation epochs. The figure shows which variables in the regional panel most frequently exhibit extreme values and how these observations are distributed across the pre-COVID, COVID, and post-COVID epochs. The largest numbers of such observations are concentrated in tariff- and cost-related indicators: internet subscription fee, z internet fee rev, and z internet fee each register 19 cases; grp total current prices registers 18; and ln internet

fee, 17. These are followed by population total (14), hospital org per 100k (13), ln grp pc (11), and ln outpatient capacity per 10k (9). For most of the remaining variables, the number of extreme values remains lower: physicians headcount records 8; nurses headcount, doctor visits total, and outpatient capacity per 10k record 7 each; and dii records 5, whereas the indicators of fixed broadband access and internet use account for only 2–3 cases, and hospital organizations count for only 1. Among the variables with the highest outlier counts, the visual distribution indicates that the post-COVID epoch contributes the predominant share of extremes. The phrasing is aligned with the dissertation's established English register for this figure and the accompanying outlier-diagnostics section
