# Supplementary material for: Digital infrastructure and proxies of ambulatory care access in Russia, 2018–2024: a regional panel study with a national telemedicine signal analysis
Source: Front Digit Health. 2026 Jun 23;8:1856577. doi: 10.3389/fdgth.2026.1856577 (PMC13338863; doi:10.3389/fdgth.2026.1856577)
Supplement: Supplementary file 2 [file Supplementaryfile2.pdf]

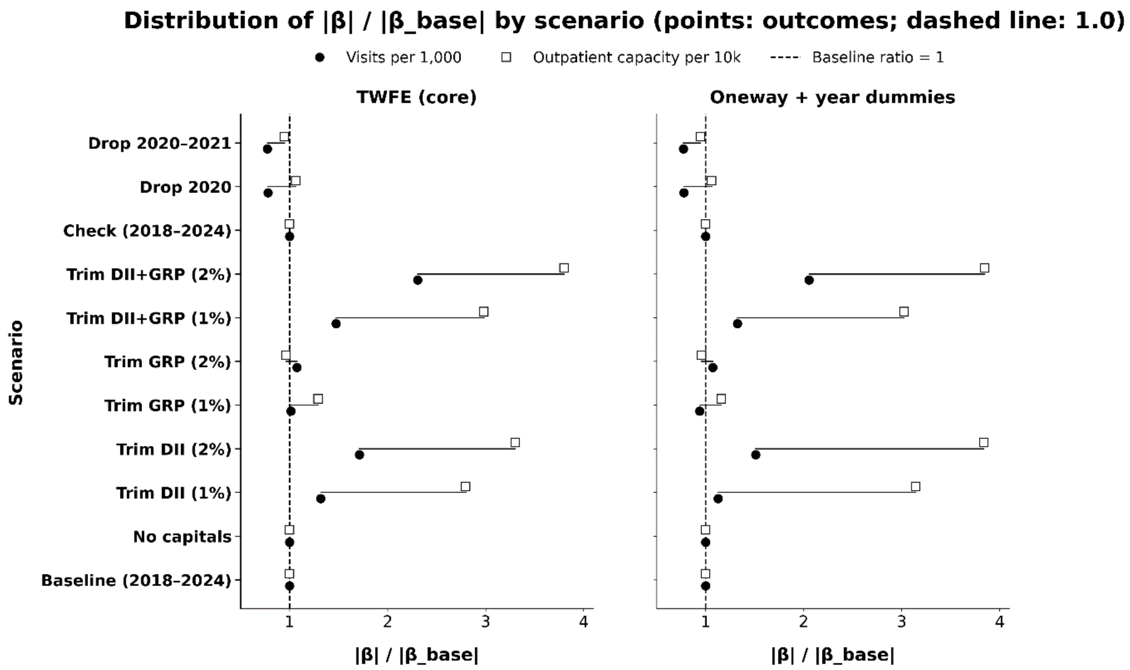

Figure S17. Sensitivity distribution across scenarios: |β|/|β_base| under TWFE (core) and one-way FE (year FE), by outcome. The figure consists of two panels: TWFE (core) on the left and one-way FE + year dummies on the right. For each scenario, the x-axis plots the ratio of the absolute coefficient magnitude to the baseline estimate, while the y-axis lists the scenarios: Baseline (2018–2024), No capitals, Trim DII (1%), Trim DII (2%), Trim GRP (1%), Trim GRP (2%), Trim DII+GRP (1%), Trim DII+GRP (2%), Check (2018–2024), Drop 2020, and Drop 2020–2021. Black filled circles correspond to the outcome Visits per 1,000, whereas open squares correspond to Outpatient capacity per 10k; in some rows, the two estimates are additionally connected by a horizontal segment. The vertical dashed line at 1.0 marks the baseline ratio. In both panels, the Baseline, No capitals, and Check scenarios lie in immediate proximity to the 1.0 line. By contrast, scenarios involving trimming on DII and on DII+GRP are displaced to the right of the baseline line, with the highest values observed for Trim DII (1–2%) and Trim DII+GRP (1–2%). Conversely, the Drop 2020 and, especially, Drop 2020–2021 scenarios are positioned to the left of the 1.0 line. The configuration of points for Visits per 1,000 and Outpatient capacity per 10k is broadly similar across the two model specifications, while the between-scenario dispersion remains visible in both the left-hand and right-hand panels. The wording is aligned with the dissertation’s established English phrasing for Figure 56, where most scenarios are described as remaining close to 1, DII-trimming scenarios as shifting the ratio above 1, and period exclusions as shifting it below 1

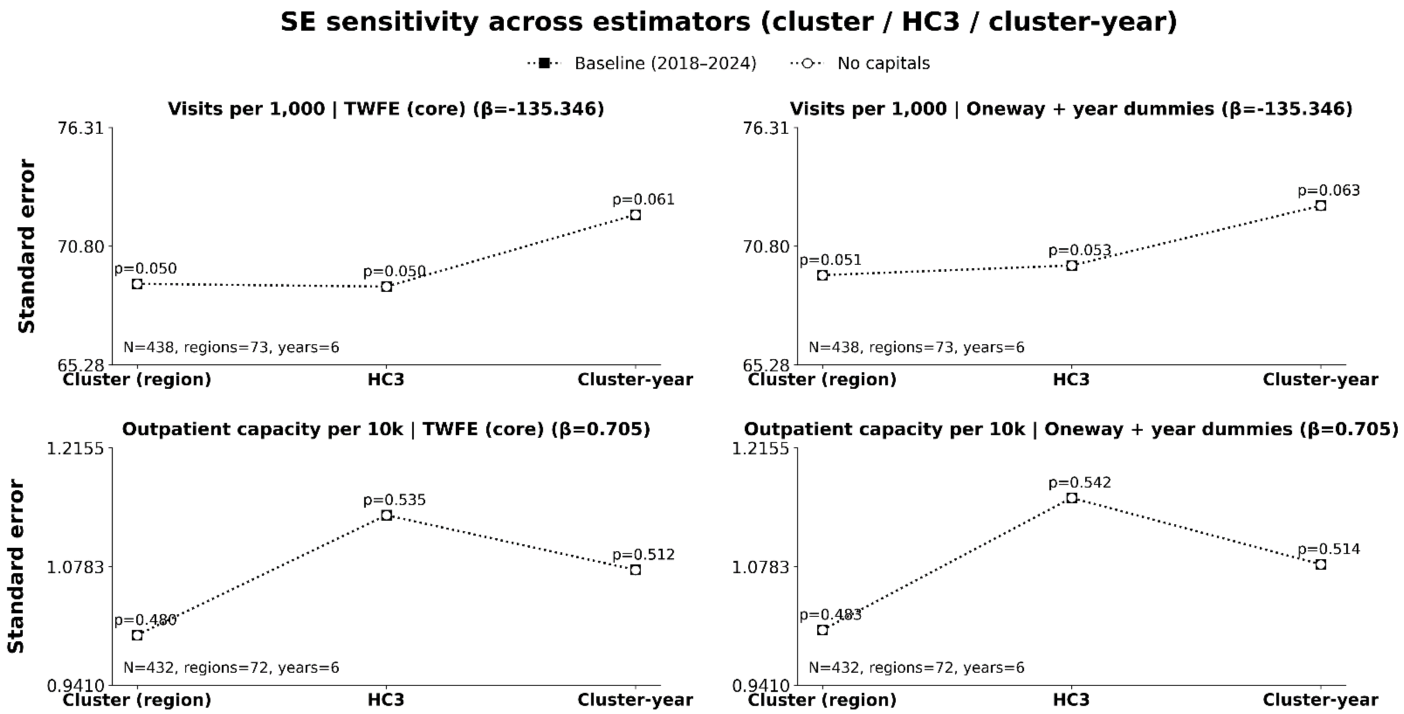

Figure S18. Sensitivity to the standard-error estimator: comparison of SE for β(DII) under Cluster(region), HC3, and Cluster(year) in TWFE and one-way FE models. he figure consists of four panels: the upper row pertains to the outcome Visits per 1,000, the lower row to Outpatient capacity per 10k; the left-hand column corresponds to the TWFE (core) specification, whereas the right-hand column corresponds to one-way FE + year dummies. Within each panel, three approaches to the estimation of the standard error are compared—Cluster (region), HC3, and Cluster-year—separately for the Baseline (2018–2024) and No capitals scenarios. For Visits per 1,000, the coefficient estimate is identical across the two specifications and equals −135.346; under TWFE (core), the corresponding p-values are 0.050, 0.050, and 0.061, whereas under one-way FE + year dummies they are 0.051, 0.053, and 0.063. In both upper panels, the sample characteristics are the same: N = 438, regions = 73, years = 6; the standard-error scale spans the interval from 65.28 to 76.31. For Outpatient capacity per 10k, the coefficient estimate is 0.705; under TWFE (core), the p-values are 0.480, 0.535, and 0.512, whereas under one-way FE + year dummies they are 0.483, 0.542, and 0.514. In the lower panels, the sample characteristics are N = 432, regions = 72, years = 6, and the standard-error scale ranges from 0.9410 to 1.2155. Across all four panels, the marker-connected lines indicate the position of the standard errors under the three estimators in the two scenarios.

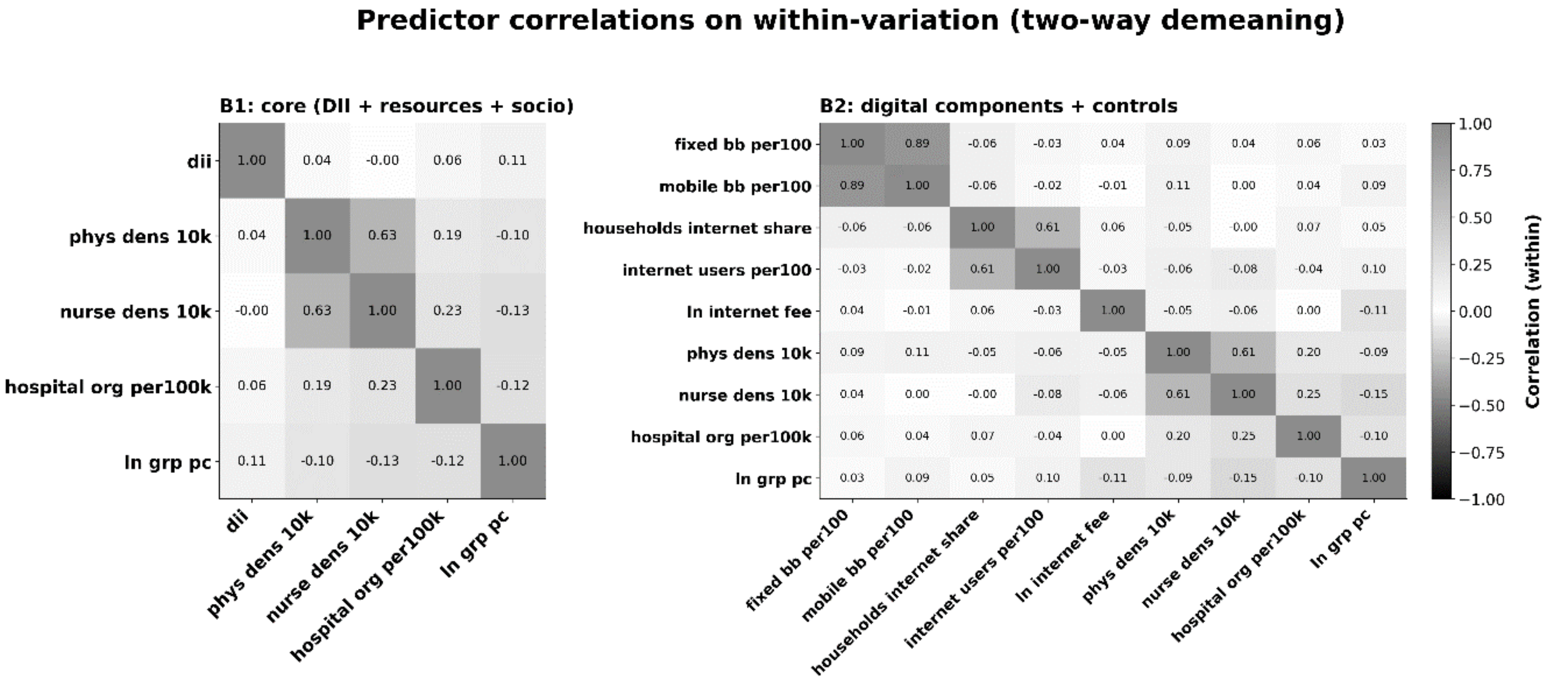

Figure 60. Matrix of within correlations among predictors after two-way demeaning (region FE and year FE). The figure presents two heatmaps that depict the correlation structure among predictors after removing time-invariant regional differences and common year-specific shifts. The left-hand panel corresponds to Block B1: core (DII + resources + socio) and includes five variables: DII, physician density, nurse density, hospital organizations per 100,000 population, and ln(GRP per capita). Within this block, the strongest positive association is observed between physician density and nurse density (0.63); the correlations of DII with the remaining components are small, lying in the range from approximately −0.00 to 0.11, while the associations of hospital org per100k and ln(GRP per capita) with the other variables remain moderate in magnitude. The right-hand panel represents Block B2: digital components + controls, comprising indicators of fixed and mobile broadband access, households internet share, internet users per100, ln internet fee, as well as the resource and socio-economic controls. In this matrix, the strongest positive correlation is recorded between fixed bb per100 and mobile bb per100 (0.89). A separate dense node is formed by households internet share and internet users per100 (0.61). Among the resource indicators, the positive association between phys dens 10k and nurse dens 10k remains evident (0.61), whereas the correlation of hospital org per100k with nurse dens 10k is 0.25 and with phys dens 10k 0.20. Most of the remaining coefficients are concentrated close to zero, predominantly within the range from −0.15 to 0.11.
