# Supplementary material for: Digital infrastructure and proxies of ambulatory care access in Russia, 2018–2024: a regional panel study with a national telemedicine signal analysis
Source: Front Digit Health. 2026 Jun 23;8:1856577. doi: 10.3389/fdgth.2026.1856577 (PMC13338863; doi:10.3389/fdgth.2026.1856577)
Supplement: Supplementary file 3 [file Supplementaryfile3.pdf]

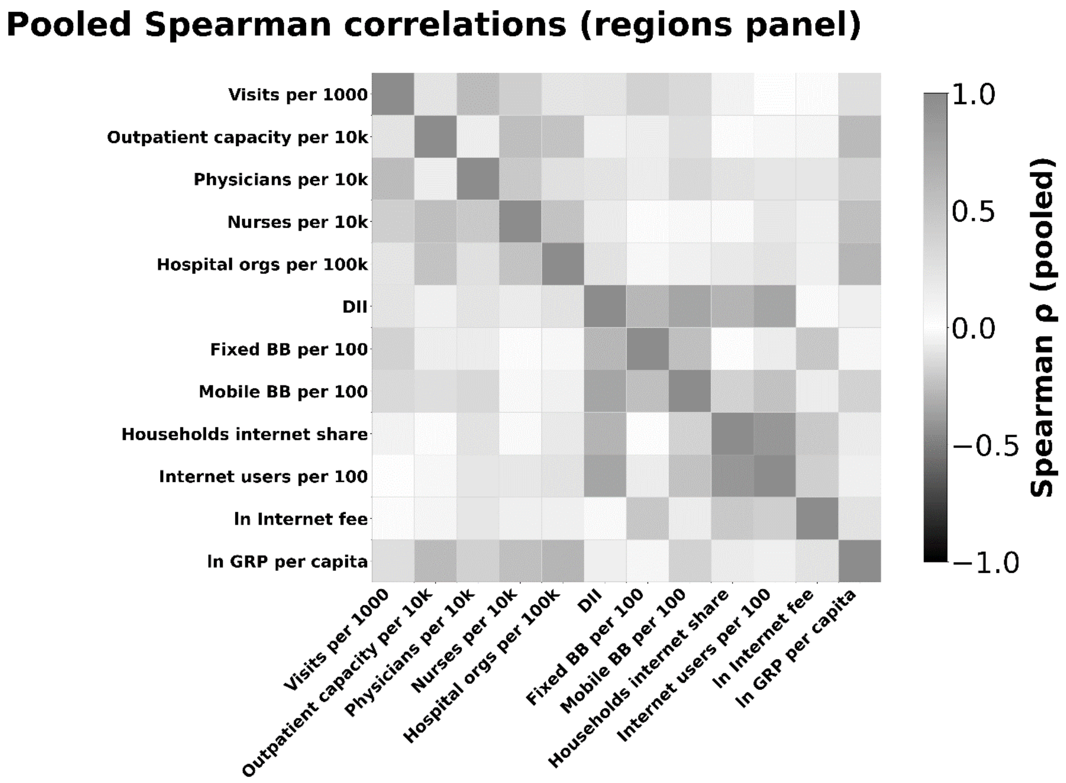

Figure S4. Pooled Spearman correlation matrix in the "Region × Year" panel, 2018–2024. The figure presents a matrix of pooled Spearman correlations for the principal outcomes, resource indicators, digital measures, and ln(GRP per capita) in the regional panel over 2018–2024. The overall configuration displays a pronounced block structure: health-system indicators form a group of predominantly positively aligned associations, whereas the digital variables and the DII constitute a more compact and denser cluster. The strongest concordance is observed within the digital block itself, where high positive associations are recorded among the DII, broadband indicators, internet use, and household internet access; it is precisely this figure that serves as the point of departure for the subsequent comparison of the pooled and within(TW) contours. Against this background, the links between the digital block and the access/load outcomes appear weaker and more heterogeneous than the within-block digital associations, although in pooled space their direction remains predominantly positive. In the dissertation text, this pattern is further specified for individual pairs: for example, the pooled association of the DII with visits per 1,000 is approximately +0.234, and with outpatient capacity per 10,000 approximately +0.129; for fixed broadband, the corresponding estimates reach +0.380 and +0.148.

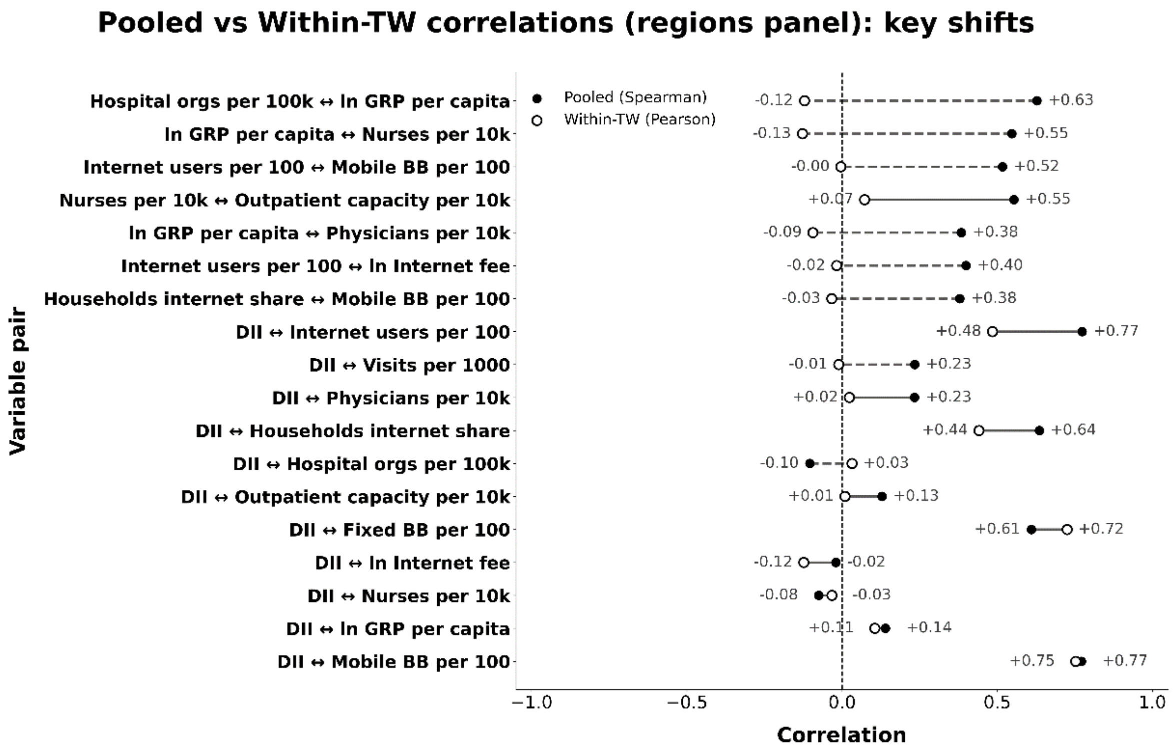

Figure S5. Slopechart comparing pooled and within(TW) correlations for selected indicator pairs, 2018–2024. The figure juxtaposes pooled Spearman correlations with within(TW) Pearson correlations after two-way demeaning for pairs linking digital indicators to access/load outcomes, as well as for associations between the DII and its constituent components. For pairs involving visits per 1,000, the pooled signal attenuates markedly once the analysis shifts to the within(TW) contour: for Fixed BB per 100 ↔ Visits per 1,000, the estimate changes from +0.380 to −0.012; for Mobile BB per 100 ↔ Visits per 1,000, from +0.319 to +0.027; and for DII ↔ Visits per 1,000, from +0.234 to −0.011. A similar profile is observed for DII ↔ Outpatient capacity per 10,000, where the association is +0.129 in pooled mode and +0.010 after two-way demeaning. Against this background, the associations between the DII and its digital components remain high even after the within(TW) transformation: DII ↔ Fixed BB per 100 is +0.72 versus +0.61; DII ↔ Mobile BB per 100, +0.77 versus +0.75; and DII ↔ Internet users per 100, +0.77 versus +0.48. The association between DII and ln GRP per capita remains moderate in both contours, at +0.14 and +0.11, respectively. The figure therefore demonstrates a sharp compression of the digital block's associations with visits and outpatient capacity when attention is restricted to within-region dynamics, whereas the alignment of the DII with its infrastructural components remains largely preserve

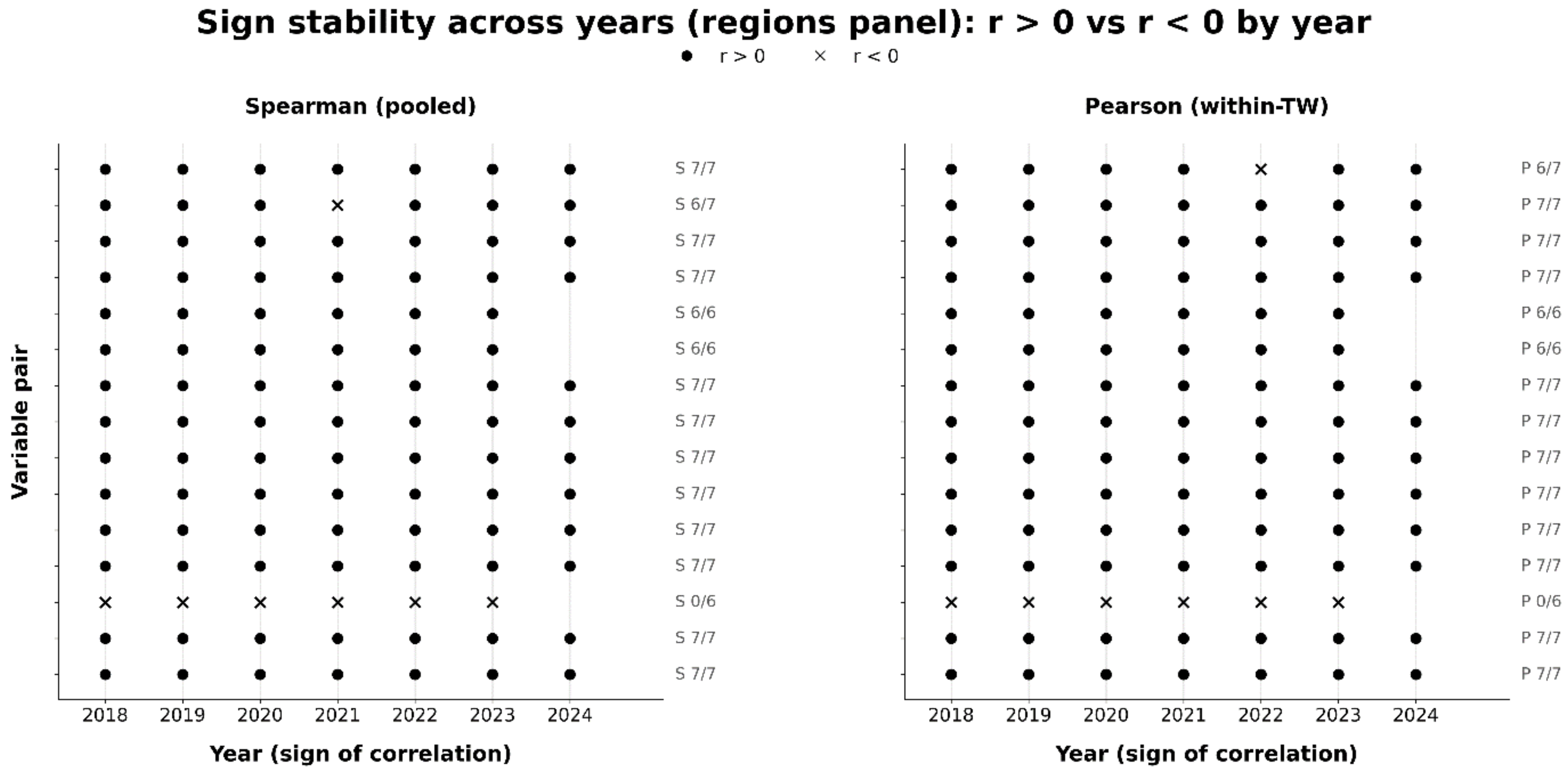

Figure S6. Year-specific correlations for selected pairs (2018–2024): pooled (Spearman) and within(TW) (Pearson). The figure displays the year-by-year reproducibility of the correlation sign for selected variable pairs across two analytical contours—pooled and within(TW). In the pooled panel, the sign is reproduced for most pairs across almost the entire observation window: in the typical case, the positive direction is retained in 6–7 of 7 years; a closely comparable pattern is also observed for the within(TW) estimates. For some pairs, only six annual data points are available, as reflected in the summary labels 6/6 and 0/6. Against this background, a negative sign remains uncommon and is concentrated in a limited number of combinations, whereas for the overwhelming majority of rows in both panels the association retains a positive direction. At the same time, the within(TW) contour exhibits more isolated sign reversals and a greater number of missing year-specific points than the pooled mode.

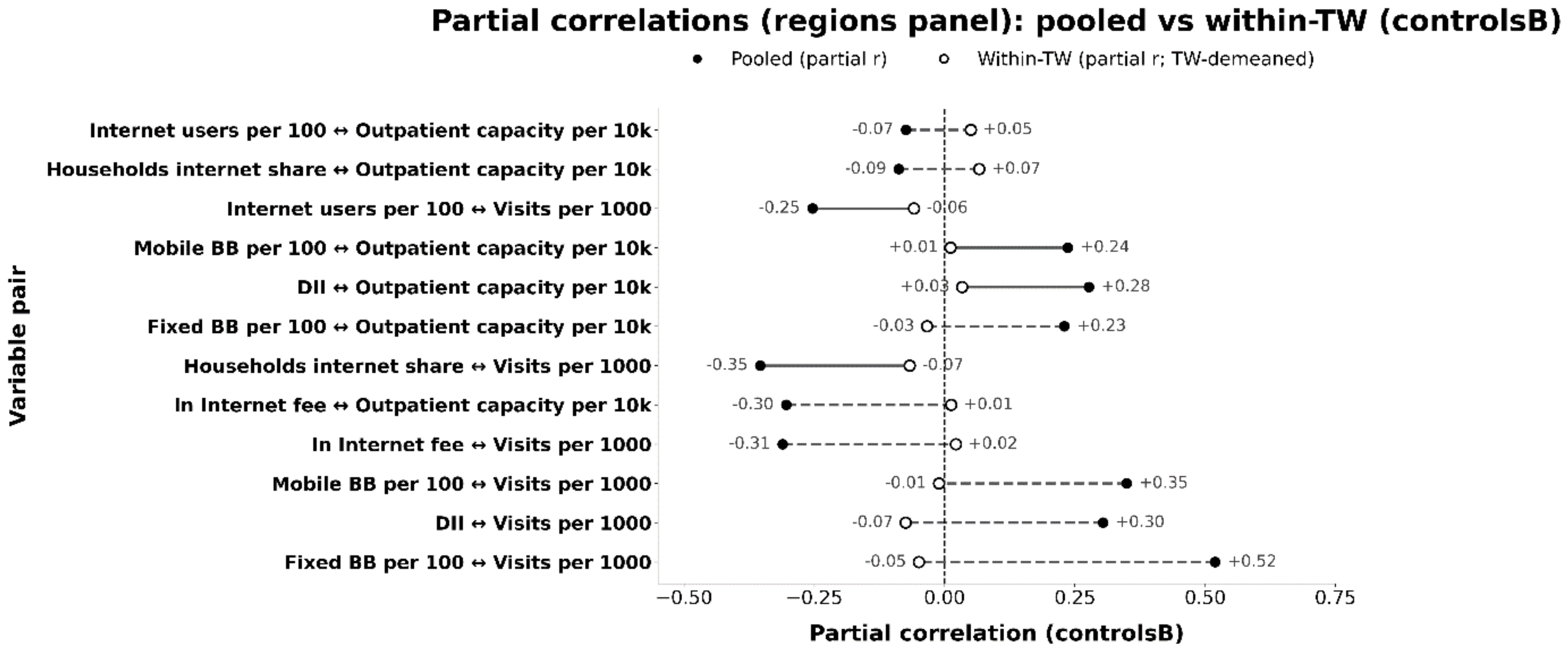

Figure S7. Partial correlations (ControlsB) for “digital indicators ↔ outcomes”: pooled (year FE) and within(TW), 2018–2024. he figure presents partial correlations between digital indicators and the two focal outcomes after adjustment for an expanded set of socio-economic and health-system resource variables. For visits per 1,000, the pooled contour retains positive estimates for Fixed BB per 100, Mobile BB per 100, and the DII, whereas in the within(TW) contour the corresponding values become slightly negative and cluster close to zero. For outpatient capacity per 10k, the positive pooled estimates likewise exceed their within(TW) counterparts: for the DII, Mobile BB, and Fixed BB, the positive pooled signal attenuates substantially once the analysis shifts to the within(TW) contour. Among variables characterised by a negative pooled profile, Households internet share ↔ Visits, Internet users ↔ Visits, and Internet subscription fee ↔ Outpatient capacity remain negative, but their absolute magnitude diminishes in the within(TW) specification; by contrast, for Internet subscription fee ↔ Visits, the sign reverses to weakly positive. For most pairs, the within(TW) estimates lie within a

narrow band around zero, whereas the pooled contour preserves a much wider dispersion of both positive and negative values. This rendering is consistent with the dissertation's established English description of Figure 37 and the accompanying interpretation of the ControlsB contour.

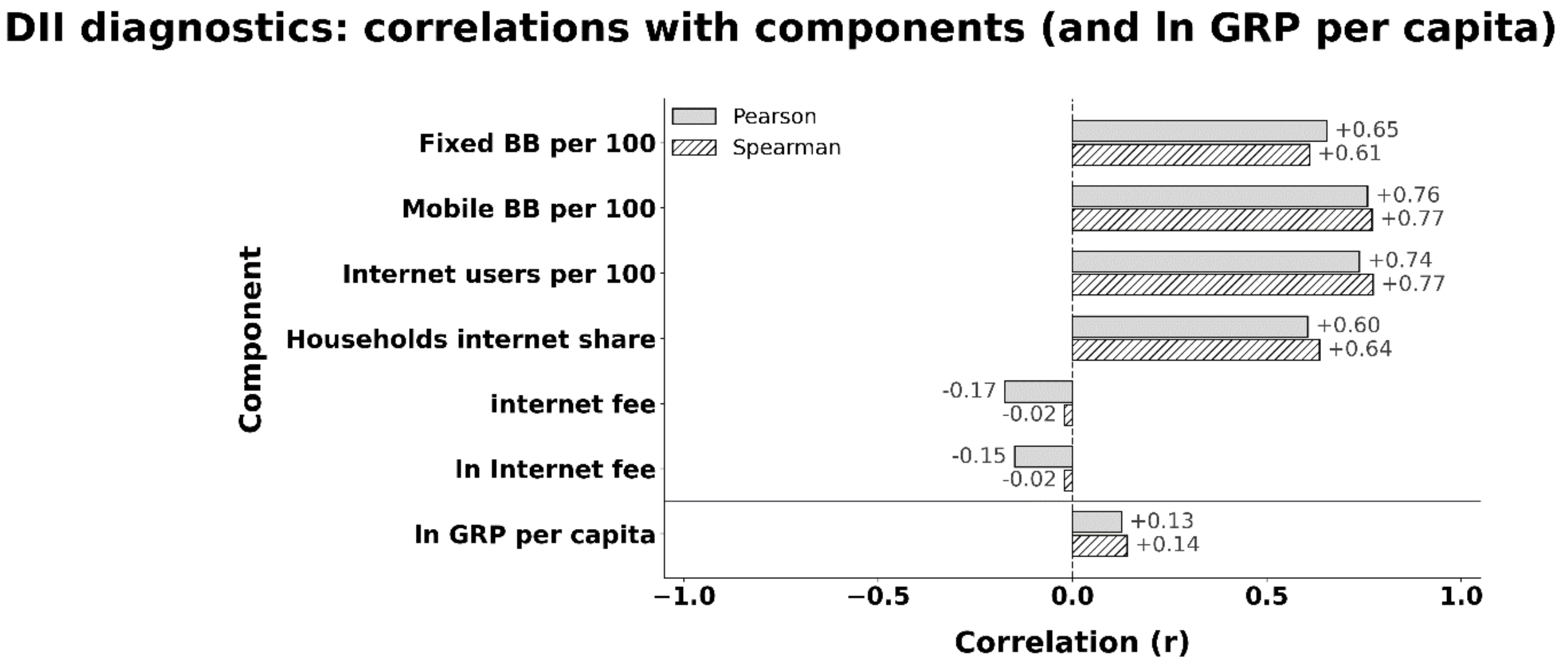

Figure S8. DII diagnostic: correlations with digital components and with ln(GRP per capita), 2018–2024. The figure presents the correlations of the DII with individual components of the digital environment and with ln(GRP per capita) under two metrics, Pearson and Spearman. The strongest positive associations are observed with Mobile BB per 100, at 0.76 by Pearson and 0.77 by Spearman; with Internet users per 100, at 0.74 and 0.77; with Fixed BB per 100, at 0.65 and 0.61; and with Households internet share, at 0.60 and 0.64. For these indicators, the discrepancy between the two coefficients is minimal, and the direction of association is consistent across both metrics. The association between the DII and ln(GRP per capita) remains substantially weaker, at 0.13 by Pearson and 0.14 by Spearman. Internet fee displays a different profile, with a Pearson correlation of −0.17 and a Spearman correlation of −0.02. Within this diagnostic contour, the DII therefore shows its closest alignment with indicators of digital infrastructure and internet use, a moderate association with regional income level, and only a weak negative association with the price-based indicator of access. The positioning

of the estimates along the correlation axis further indicates that the digital components form a compact cluster in the approximate range from 0.60 to 0.77, whereas Internet fee and ln(GRP per capita) occupy a more isolated position closer to zero.

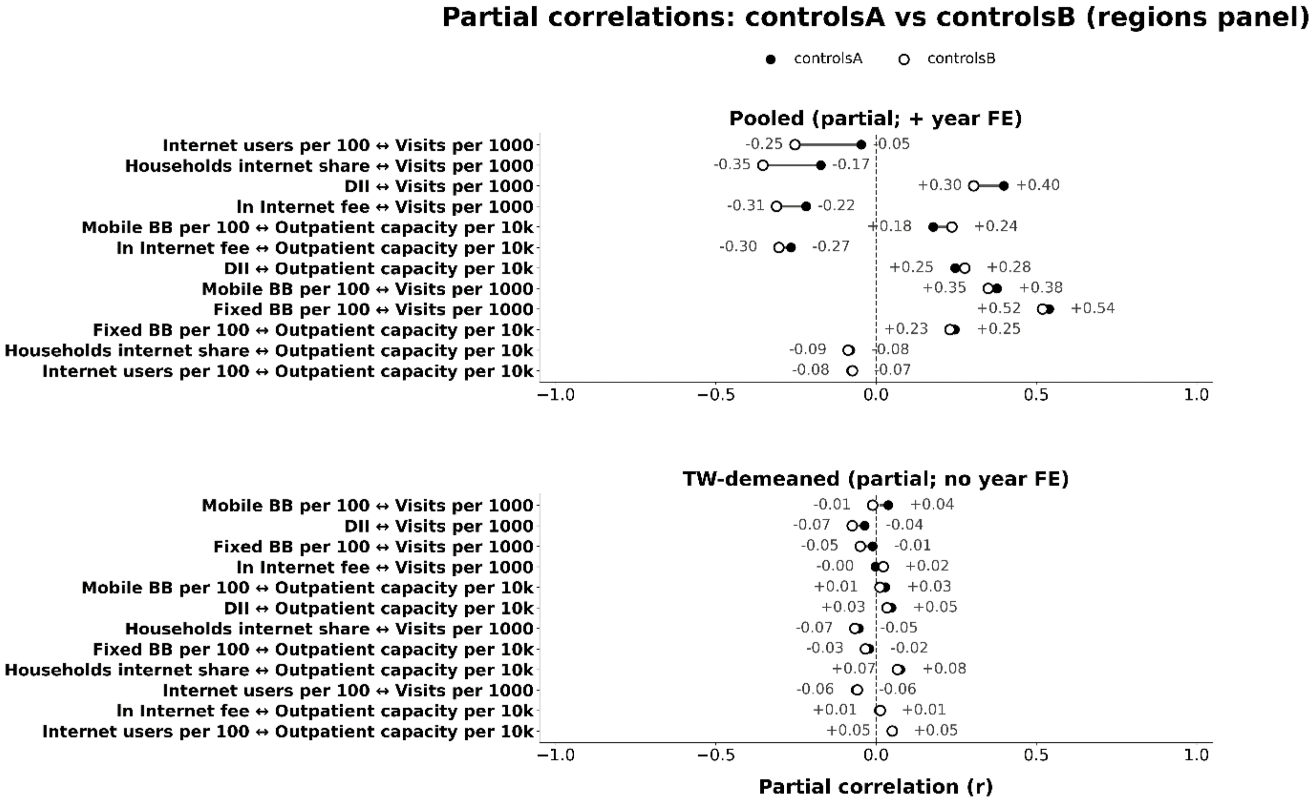

Figure S9. Partial correlations under alternative control sets (ControlsA vs ControlsB) in pooled and within(TW) specifications, 2018–2024. The figure compares partial correlations for six digital-indicator–outcome pairs under two alternative control specifications. In the upper panel, corresponding to the pooled estimates, all associations remain positive under both control sets. For visits per 1,000, the values are 0.40 under ControlsA and 0.30 under ControlsB for the DII; 0.54 and 0.52 for Fixed BB per 100; and 0.38 and 0.35 for Mobile BB per 100. For outpatient capacity per 10k, the corresponding pairs yield 0.25 and 0.28 for the DII; 0.25 and 0.23 for Fixed BB per 100; and 0.18 and 0.24 for Mobile BB per 100. In the lower panel, corresponding to the within(TW) estimates, all values lie close to zero. For visits per 1,000, the DII

yields −0.04 under ControlsA and −0.07 under ControlsB; Fixed BB per 100, −0.01 and −0.05; and Mobile BB per 100, 0.04 and −0.01. For outpatient capacity per 10k, the estimates are 0.05 and 0.03 for the DII; −0.02 and −0.03 for Fixed BB per 100; and 0.03 and 0.01 for Mobile BB per 100. Taken together, the figure indicates that the pooled contour preserves a stable positive profile under both control regimes, whereas the within(TW) contour compresses the same associations into a narrow near-zero range, with only minor sensitivity to the choice between ControlsA and ControlsB. This rendering is aligned with the dissertation's established English phrasing for Figure 39 and its interpretation of the contrast between pooled and within(TW) residual associations.

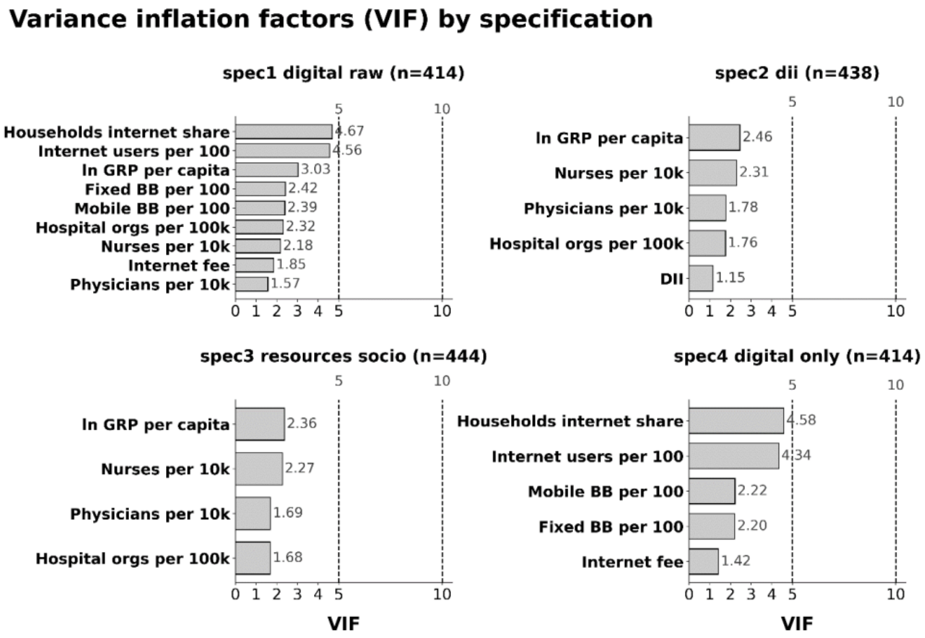

Figure S10. Distribution of VIF across model specifications (thresholds 5 and 10), 2018–2024. The figure displays the variance inflation factor values for four predictor sets, with threshold lines at 5 and 10. In the digital raw specification, based on 414 observations, the highest values are observed for Households internet share (4.67) and Internet users per 100 (4.56); these are followed by ln GRP per capita (3.03), Fixed BB per 100 (2.42), Mobile BB per 100 (2.39), Hospital orgs per 100k (2.32), Nurses per 10k (2.18), Internet fee (1.85), and Physicians per 10k (1.57). In the dii specification, based on 438 observations, the values fall within a narrower range: ln GRP per capita

reaches 2.46, Nurses per 10k 2.31, Physicians per 10k 1.78, Hospital orgs per 100k 1.76, and the DII 1.15. In the resources socio set, based on 444 observations, the highest values are 2.36 for ln GRP per capita and 2.27 for Nurses per 10k, while all remaining indicators stay below 1.70. In the digital only specification, again based on 414 observations, Households internet share (4.58) and Internet users per 100 (4.34) once more stand out, whereas Mobile BB per 100 and Fixed BB per 100 are 2.22 and 2.20, respectively, and Internet fee is 1.42. Across all four sets, the maximum values remain below the threshold line of 5.

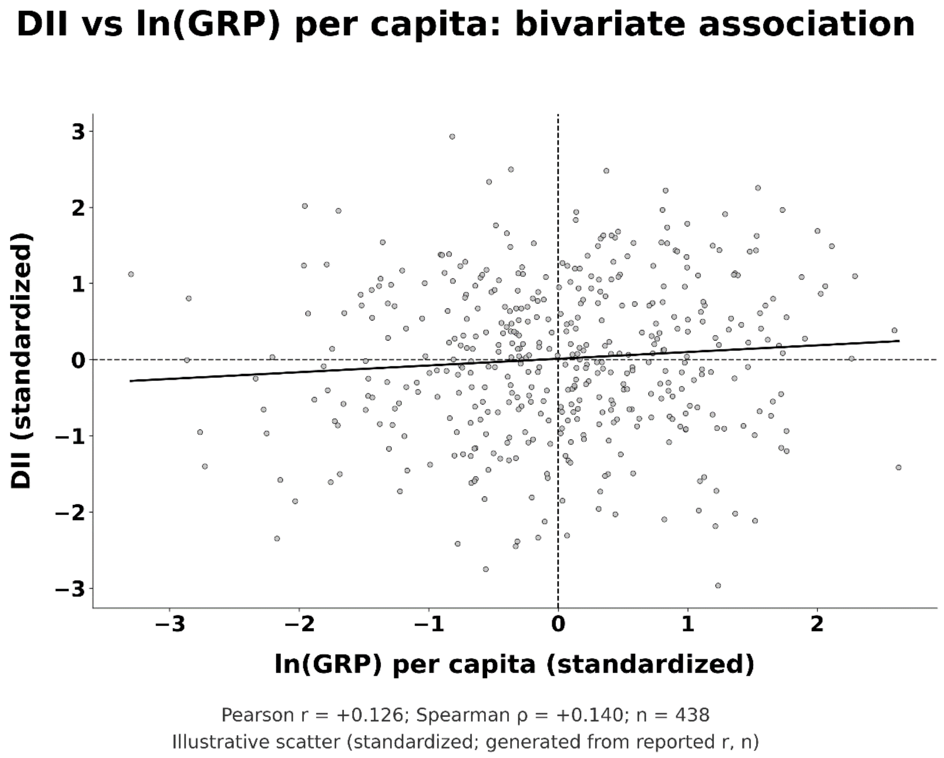

Figure S11. Bivariate association between DII and ln(GRP per capita): standardized scatter with a linear fit. The figure presents a standardised scatterplot for the DII and ln(GRP per capita) with a superimposed linear fit. In both metrics, the association remains positive yet weak: the Pearson coefficient is 0.126, the Spearman coefficient 0.140, and the number of observations 438. Most points are concentrated around the central zone of the standardised scale; at the same time, the point cloud remains broad

along both axes and is only faintly elongated in an upward direction. The fitted line corresponds to a modest positive gradient, although no tight clustering of observations around it is evident. Along the x-axis, ln(GRP per capita) extends approximately from −3 to +2 standard deviations, whereas along the y-axis the DII ranges from approximately −3 to +3. As indicated in the figure caption, what is shown is an illustrative standardised cloud generated from the reported correlation values and the sample size. The figure should therefore be read as conveying the direction of the association, its overall magnitude, and its degree of dispersion, rather than as inviting any literal reading of individual points as original observations from the underlying dataset. This wording is aligned with the dissertation's established English phrasing for Figure 41, where the relationship is described as weakly positive amid wide interregional dispersion and as supporting an interpretation of the DII as not directly reducible to regional economic development level.
