# Supplementary material for: Digital infrastructure and proxies of ambulatory care access in Russia, 2018–2024: a regional panel study with a national telemedicine signal analysis
Source: Front Digit Health. 2026 Jun 23;8:1856577. doi: 10.3389/fdgth.2026.1856577 (PMC13338863; doi:10.3389/fdgth.2026.1856577)
Supplement: Supplementary file 4 [file Supplementaryfile4.pdf]

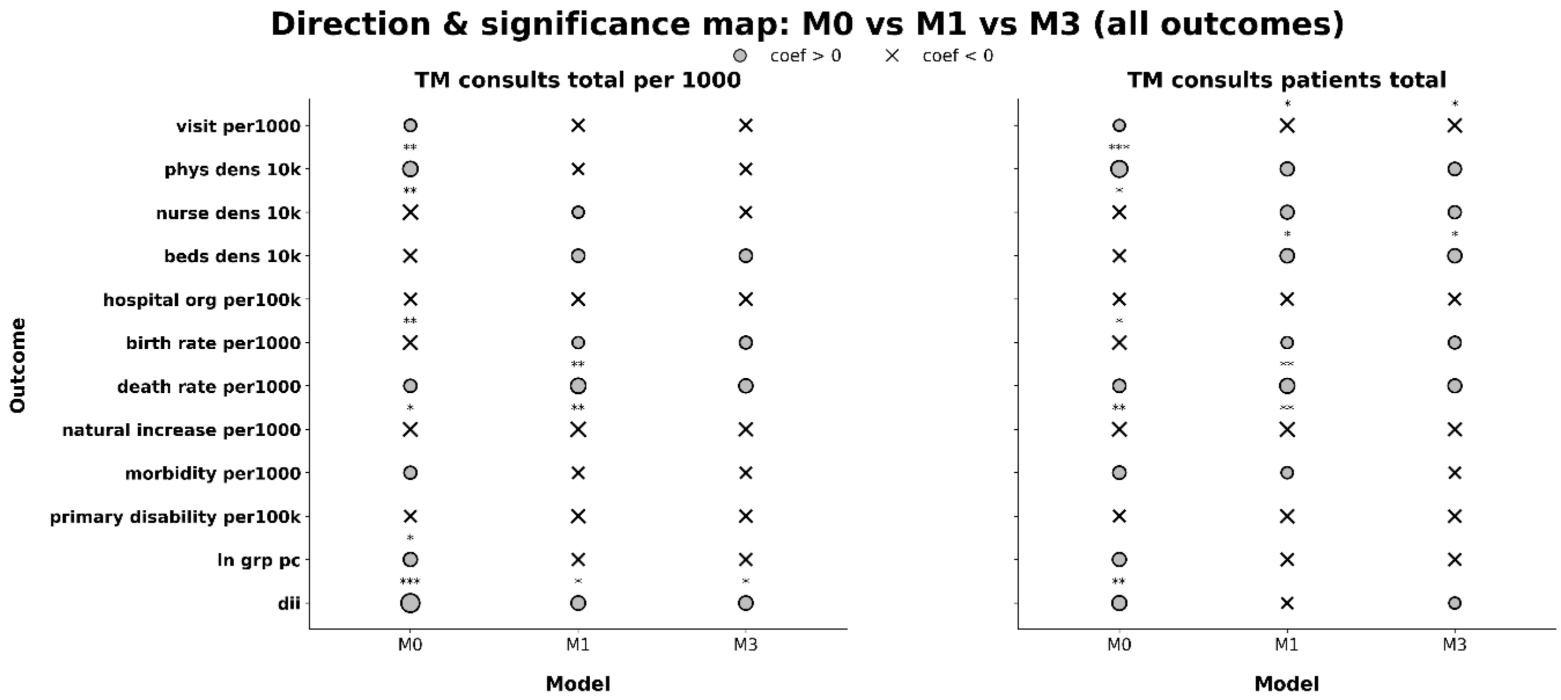

Figure S12. RF–year: map of effect direction and statistical support for β estimates linking TM × outcome in M0, M1, and M3 (separately for total exposure and the "patient" component). The figure displays the sign of the estimated association and the corresponding level of statistical support for telemedicine coefficients across three model forms—levels (M0), levels with a linear trend (M1), and first differences (M3)—shown separately for total telemedicine consultations per 1,000 population and for patient telemedicine consultations. In the panel for total consultations, DII remains positive across all three specifications and retains statistical support in each case, from three stars in M0 to one star in M1 and M3. Death rate per 1,000 also remains positive throughout, with support most visible in M1, whereas natural increase per 1,000, hospital organizations per 100,000, and primary disability per 100,000 remain negative in all three models. By contrast, several outcomes change sign between specifications, including visits per 1,000, physician density, nurse density, beds per 10,000, birth rate, morbidity, and ln(GRP per capita). In the panel for patient telemedicine consultations, the sign profile is similar for some outcomes: hospital organizations, natural increase, and primary disability remain negative, while death rate stays positive. Visits per 1,000 are positive in M0 and negative in both M1 and M3. DII is positive and statistically supported in M0, negative in M1, and positive again in M3.

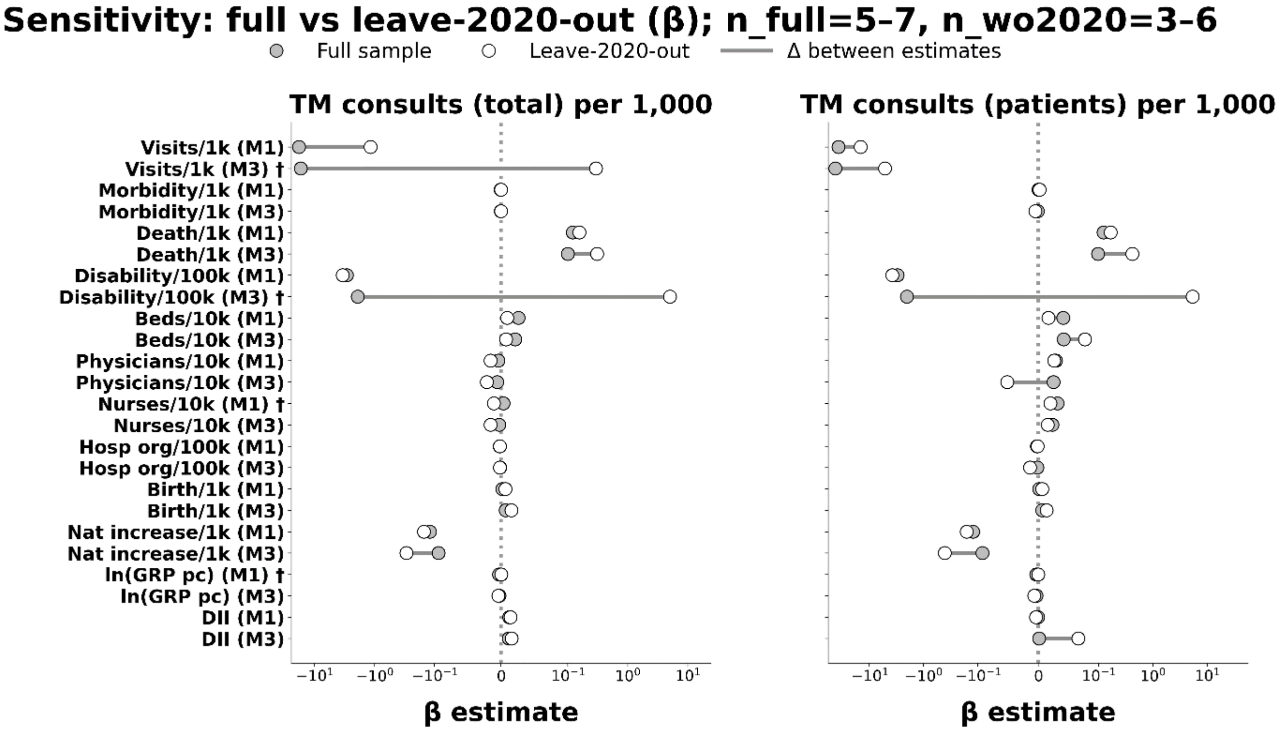

Figure S13. RF–year: comparison of β estimates in the full sample vs excluding 2020 for specifications M1 and M3 (two TM operationalizations). The figure compares β estimates obtained from the full sample with those re-estimated after excluding 2020 for models M1 and M3, using two alternative telemedicine indicators—total per 1,000 and patients per 1,000. The horizontal segments represent the discrepancy between the two estimates, while the number of observations ranges from 5–7 in the full sample to 3–6 once 2020 is omitted. The x-axis is presented on a symmetric logarithmic scale, such that the contrasts primarily reflect shifts in order of magnitude and direction. In both panels, the most pronounced departures are observed for Visits/1k and Disability/100k, particularly in M3: for Visits/1k in the left panel, the full-sample estimate lies in the negative range, whereas the estimate obtained without 2020 shifts into the positive range; for Disability/100k, one of the longest intervals between the two estimates is likewise visible in M3 across both panels. For Death/1k, under both M1 and M3, the estimates remain in the positive part of the scale and vary more modestly. For Beds/10k, Physicians/10k, Nurses/10k, and Hosp org/100k, most estimates are concentrated closer to the central zone. For Nat increase/1k, both estimates in both panels remain in the negative range. By contrast, for ln(GRP pc) and DII, the visual profile suggests more moderate displacement relative to Visits/1k and Disability/100k.

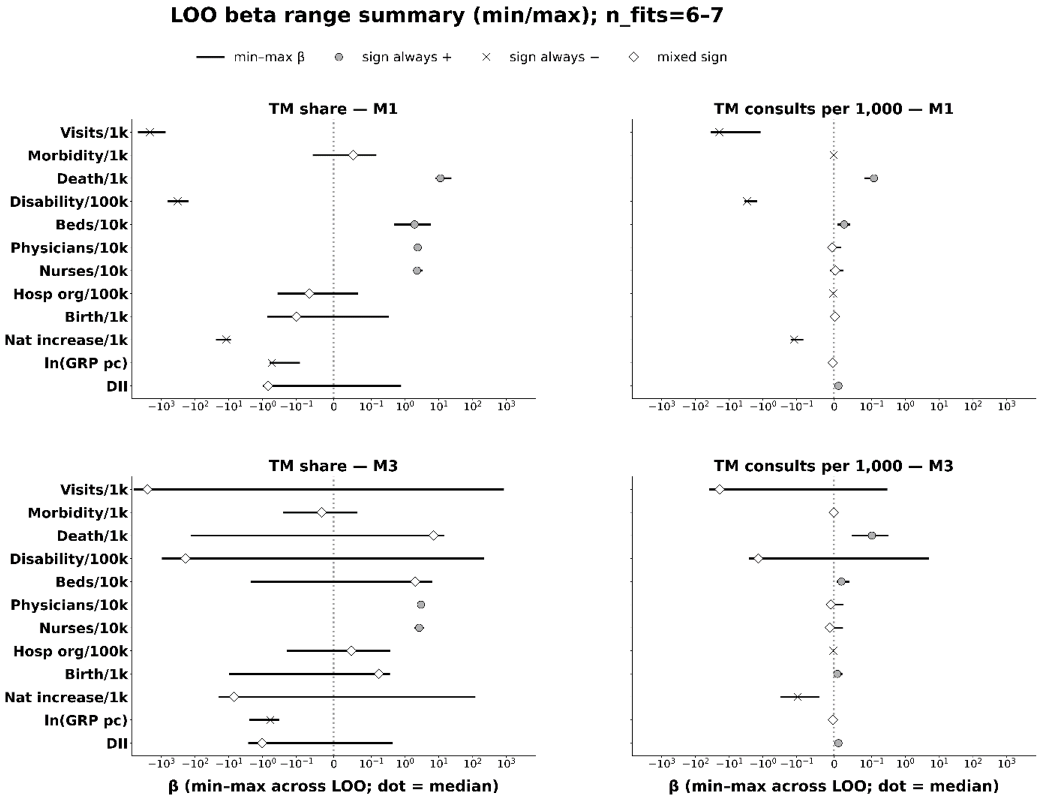

Figure S14. RF–year: LOO robustness of β estimates—min–max range and median across leave-one-year-out runs in M1 and M3 (two TM metrics). The figure presents the ranges of β estimates across all leave-one-year-out runs for four panels: TM share in M1, TM consults per 1,000 in M1, TM share in M3, and TM consults per 1,000 in M3. For each row, the horizontal segment denotes the interval from the minimum to the maximum value, the point marks the median, and the symbol indicates the sign pattern across all LOO runs: consistently positive, consistently negative, or mixed. In the TM share—M1 panel, a consistently positive sign is observed for Death/1k, Beds/10k, Physicians/10k, and Nurses/10k, whereas a consistently negative sign is observed for Visits/1k, Disability/100k, Nat increase/1k, and ln(GRP pc); Morbidity/1k, Hosp org/100k, Birth/1k, and DII, by contrast, fall into the mixed-sign profile. In the TM consults per 1,000—M1 panel, a positive sign is preserved for Death/1k, Beds/10k, and DII, while a negative sign is preserved for Visits/1k, Morbidity/1k, Disability/100k, Hosp org/100k, and Nat increase/1k; Physicians/10k, Nurses/10k, Birth/1k, and ln(GRP pc) display a mixed sign pattern. In the TM share—M3 panel, most outcomes belong to the mixed-sign profile; a consistently positive sign is retained only for Physicians/10k and Nurses/10k, whereas a consistently negative sign is retained only for ln(GRP pc).

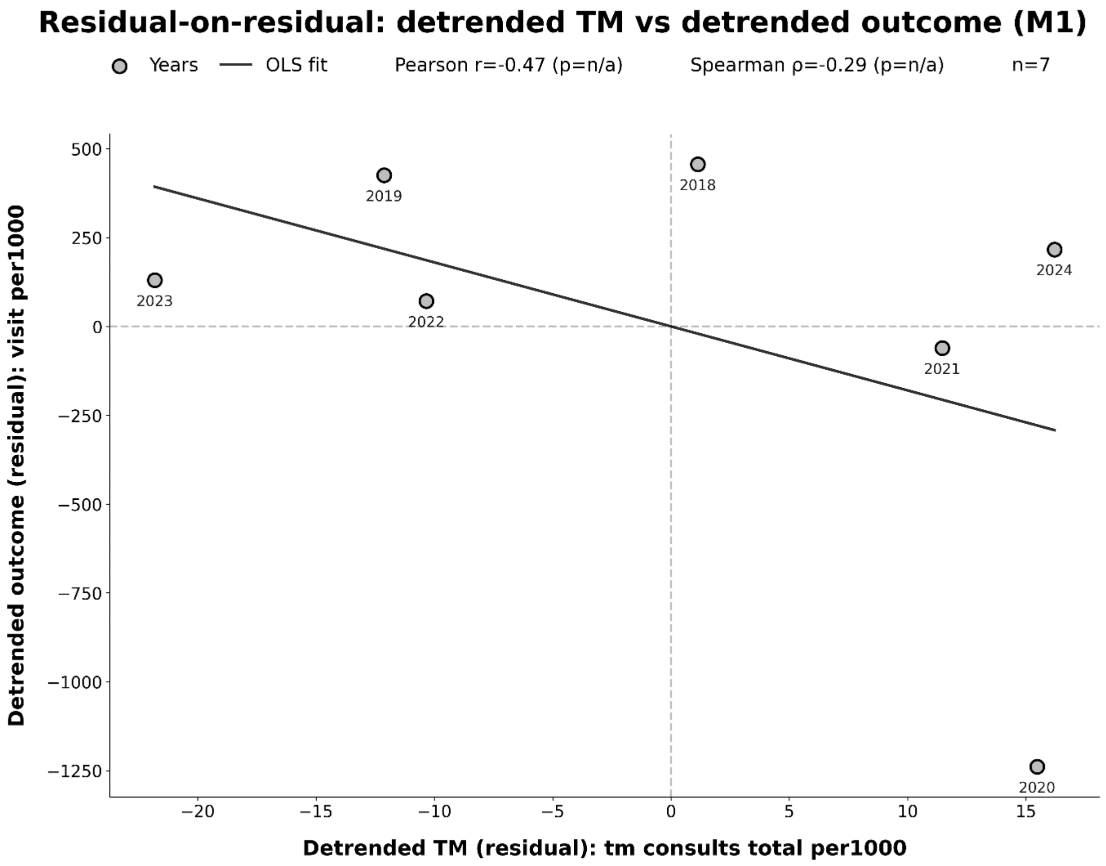

Figure S15. Residual-on-residual (M1): detrended association between telemedicine and the outcome at the RF–year level. he figure displays the relationship between the residual deviations of telemedicine consultations, total per 1,000, and visits per 1,000 after removal of the linear time trend; each point corresponds to a single observation year, and a linear fit is superimposed on the point cloud. On the coefficient scale, the association between the two residual series is negative in direction: the Pearson coefficient is −0.47, the Spearman coefficient −0.29, and the number of observations 7. The distribution of points across years remains heterogeneous. The most distant observation is 2020, which combines a markedly positive residual for the telemedicine indicator with the most pronounced negative residual for visits per 1,000. In 2021, a positive residual in telemedicine exposure is likewise paired with a negative residual in the outcome, although on a smaller scale. By contrast, 2018 and 2024 are located in the region of positive residuals on both axes.

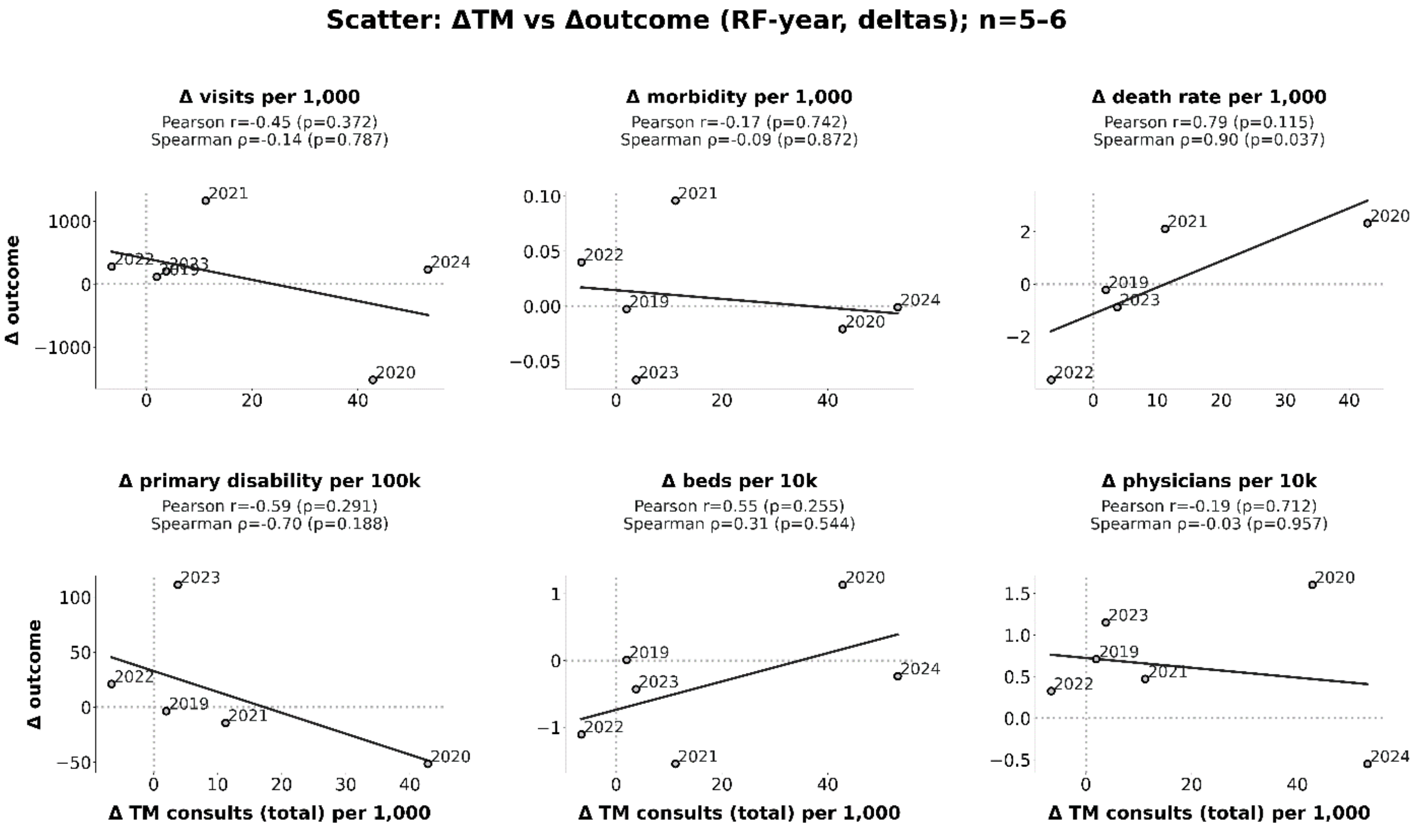

Figure S16. First differences: scatter comparison of ΔTM and Δoutcomes (RF–year). The figure presents six scatterplots in which the x-axis shows the change in TM consults, total per 1,000, while the y-axis shows the corresponding annual change in the outcome; each point is labelled by year, and the number of observations in the individual panels ranges from 5 to 6. For Δvisits per 1,000, the association is negative in Pearson terms, against an almost null rank-based profile, with coefficients of −0.45 and −0.14, respectively. For Δmorbidity per 1,000, both estimates are likewise weakly negative, at −0.17 by Pearson and −0.09 by Spearman. The most pronounced positive configuration is observed for Δdeath rate per 1,000, where the coefficients reach 0.79 and 0.90; for the rank correlation, p = 0.037 is reported. For Δprimary disability per 100k, both estimates are negative and more substantial in absolute magnitude than in the visits and morbidity panels, at −0.59 by Pearson and −0.70 by Spearman. For Δbeds per 10k, the association is positive, although its magnitude differs across the two metrics, at 0.55 and 0.31. In the Δphysicians per 10k panel, both estimates remain close to zero, at −0.19 by Pearson and −0.03 by Spearman. Across all six panels, the year labels indicate that the point clouds are formed by observations from 2019–2024.
